# Cross‐Correlation Between ^11^B Quadrupole and ^11^B‐^19^F Dipole–Dipole Coupling in BF_2_ Groups

**DOI:** 10.1002/mrc.5507

**Published:** 2024-12-20

**Authors:** Franziska Rüttger, Dominik Franke, Jannik Probst, Xiaobai Wang, Dietmar Stalke, Michael John

**Affiliations:** ^1^ Institut für Anorganische Chemie Georg‐August‐Universität Göttingen Göttingen Germany

**Keywords:** cross correlation, dipolar coupling, electric field gradient, NMR spectroscopy, quadrupolar coupling

## Abstract

We investigate cross‐correlation between ^11^B quadrupole and ^11^B‐^19^F dipole–dipole coupling in two BODIPY compounds and one bis(benzoxazol)methanide in partially oriented polystyrene (PS) samples. Especially for the bis(benzoxazol)methanide, the transitions for which the two interactions interfere con‐ or destructively clearly show distinct linewidths.

## Graphical abstract

Cross‐correlation between ^11^B quadrupole and ^11^B‐^19^F dipole–dipole coupling in BODIPY‐type compounds leads to markedly different linewidth in ^11^B NMR spectra recorded in partially oriented polystyrene (PS) samples.

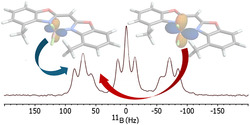

## 1 Introduction

Cross‐correlation (or relaxation interference) between different spin interactions is a long‐known effect that leads to differential linewidths and relaxation behavior in NMR multiplets [1]. The best‐known example is probably cross‐correlation between dipolar coupling and chemical shift anisotropy, which is frequently exploited in biomolecules to enhance peak resolution of amide [2] or methyl groups [3] or to obtain angular structural information [4]. However, also interference between other spin interactions—including the quadrupole interaction—is known [5], and an early example of dipolar/quadrupole cross‐correlation was given by CHDCl_2_ in liquid crystalline phase [6]. Cross‐correlation effects are expected to be large if the contributing spin interactions are (i) of similar magnitude and (ii) structurally highly correlated, that is, with a similar dependence on molecular orientation. This makes them attractive probes of unknown spin interactions if the other spin interaction is known.

**FIGURE 1:**
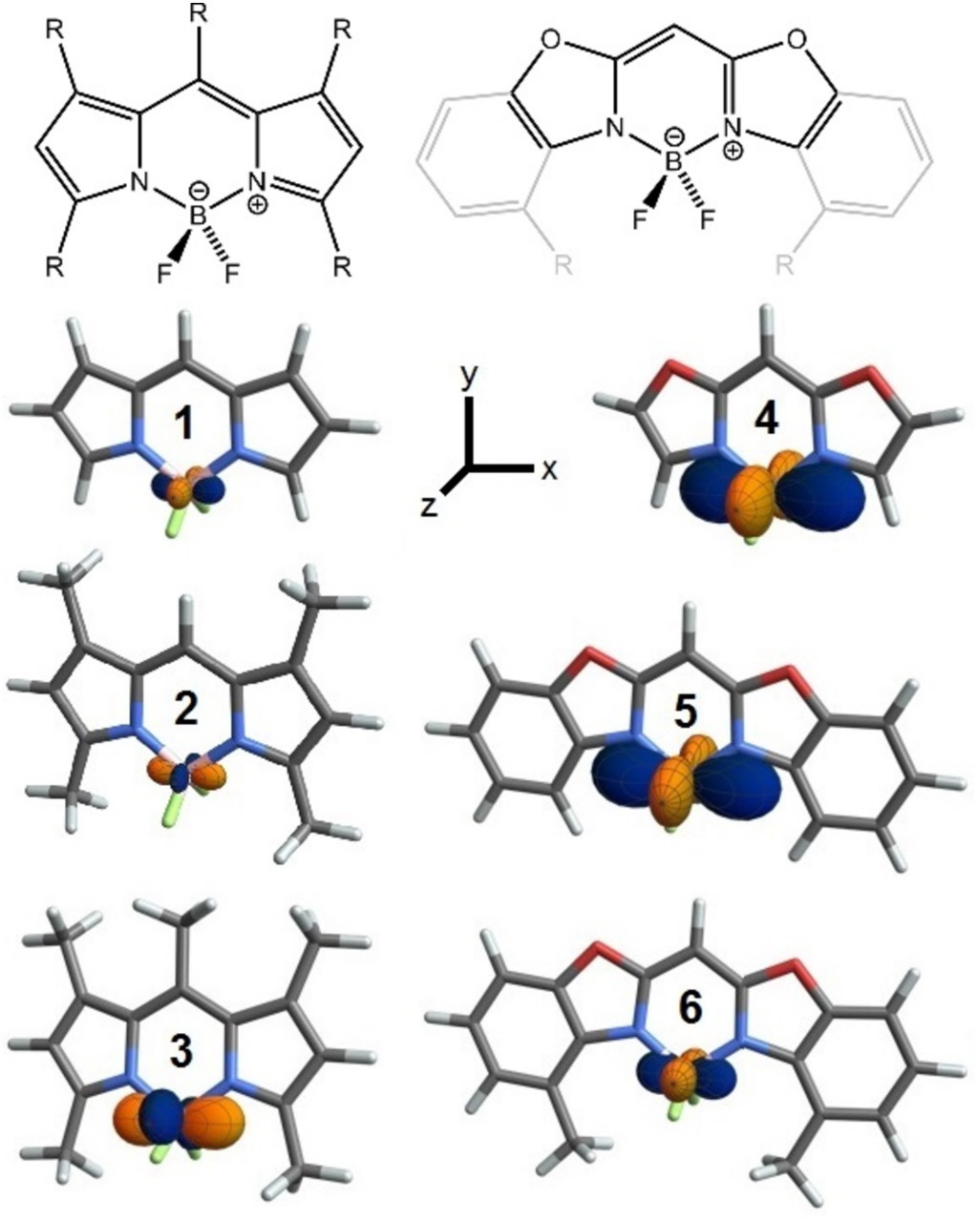
Chemical drawings (R = H, CH_3_) and DFT geometry‐optimized structures (dark grey = carbon, light grey = hydrogen, pink = boron, blue = nitrogen, red = oxygen) of selected BODIPY compounds **1**–**3** and boron bis(oxazole)methanides **4**–**6**. Calculated boron EFG tensors are displayed graphically at the boron atoms (orange = direction of negative EFG, blue = directions of positive EFG), and the coordinate system used throughout the manuscript is given.

As part of our ongoing project on ^7^Li and ^11^B residual quadrupolar couplings (RQCs) [7], we investigate cross‐correlation between ^11^B quadrupole and ^11^B‐^19^F dipole–dipole spin interactions in two types of compounds bearing BF_2_ groups as part of an indacene scaffold: BODIPYs (= boron dipyrromethenes) that have gained tremendous significance as fluorescence labels of biomolecules [8], and bis(oxazol)methanides, the dehydrogenated and deprotonated form of the well‐known BOXs (= bis(oxazolines) used in asymmetric catalysis [9] (Figure 1). Due to the near‐tetrahedral geometries at the boron atoms, the boron electric field gradients (EFGs) in both types of molecules are very small, such that the magnitudes of both spin interactions are comparable.

Using model compounds **1**–**6**, we first show that the boron EFGs are highly sensitive to small variations such as methyl substitution and then calculate the spatial dependence of both spin interactions. Cross‐correlation effects—in the form of differential linewidths in residual quadrupolar and ^19^F‐coupled ^11^B multiplets in weakly oriented samples—are experimentally demonstrated for commercial BODIPY compounds **2** and **3**, and for the boron bis(oxazol)methanide **6** that was recently synthesized in our laboratory [10].

## 2 Results and Discussion

### 2.1 Geometries

Geometries of **1**–**6** were optimized, and boron EFGs calculated using standard density functional theory (DFT) methods (Supporting Information page S2). Geometric and EFG parameters are summarized in Table 1.

**TABLE 1:** Boron bond lengths (BN, BF), bond angles (NBN, FBF), and EFG components in geometry‐optimized models of **1**–**6**.

Parameter	1	2	3	4	5	6
BN (Å)	1.568	1.561	1.550	1.562	1.562	1.565
BF (Å)	1.385	1.394	1.396	1.385	1.385	1.389
NBN (°)	105.7	106.7	106.5	103.8	104.2	106.0
FBF (°)	111.2	110.2	109.9	110.8	110.7	111.3
*V*_xx_ (10^−3^ a.u.)^a^	9.2	−9.8	−16.2	22.4	23.0	12.2
*V*_yy_ (10^−3^ a.u.)^a^	0.9	1.9	−0.2^b^	−3.9	−4.2	2.5
*V*_zz_ (10^−3^ a.u.)^a^	−10.1	7.9	16.4^b^	−18.5	−18.8	−14.8

^a^ 1 a.u. = 9.717 × 10^−21^ Vm^−2^.

^b^ Due to slight butterfly folding, the FBF bisector leaves the molecular plane and the off‐diagonal element *V*_yz_ = 2.2 × 10^−3^  a.u. becomes nonzero.

All molecules are flat or almost flat with little distortion from *C*_2v_ symmetry and generally show only minor deviations of bond lengths (< 0.01 Å) and angles (< 1°) from crystal structures that exist of **1** [11], **2** [12], **3** [13], and **6** [10]. Compounds **4** and **5** are unstable yet unknown and were only included in our analysis as “parents” of the recently isolated compound **6**. Also within the models **1**–**6**, all parameters are in a narrow range (BN = 1.550–1568 Å, BF = 1.385–1.396 Å, NBN = 103.8–106.7° = slightly below tetrahedral, FBF = 109.9–111.3° = slightly above tetrahedral). Throughout this manuscript, we use a uniform coordinate system with x = N … N, y = *C*_2_, and z = F … F directions, respectively.

### 2.2 ^11^B Quadrupole Coupling

^11^B quadrupole coupling is determined by

$$\chi =\frac{\mathrm{eQ}}{h}V$$

(**χ** = quadrupolar coupling tensor, *e* = elementary charge, *eQ* = nuclear quadrupole moment of ^11^B, *h* = Planck's constant, **V** = boron EFG tensor, for ^11^B *eQ*/*h* = 9.673 MHz/a.u.) [14]. To first order approximation (|**χ**| << ^11^B Larmor frequency) quadrupole coupling in spin‐3/2 nuclei leads to a (orientation‐dependent) frequency shift of **Δν**_**Q**_ = +**χ**/2 for the *m*_B_ = (−1/2 → –3/2) transition and of **Δν**_**Q**_ = −**χ**/2 for the *m*_B_ = (+3/2 → +1/2) transition, while the central *m*_B_ = (+1/2 → –1/2) transition remains unaffected.

Due to the near‐tetrahedral coordination of the boron atoms in **1**–**6**, the EFGs are very small (9.8–23.0 × 10^−3^ a.u. for the largest component of **V**, corresponding to ^11^B quadrupole coupling constants χ_max_ of 95–222 kHz). In fact, the EFG contributions from each type of donor atom (N and F) at near‐tetrahedral donor‐B‐donor angles can be thought of as being close to fully rhombic with small *V*_yy_ but opposite *V*_xx_ and *V*_zz_ that tend to cancel each other. Because F is in the spectrochemical series generally regarded as both good σ (generating negative boron EFG along the B‐F bond) and π donor (generating negative EFG perpendicular to the B‐F bond) [15], the total EFG is very sensitive to the corresponding σ(N) donor and π(N) donor/acceptor properties of the dipyrromethene or bis(oxazole)methanide ligand. These are in turn related to important dye properties (optical absorption/emission maxima) and may be estimated from molecular orbital calculations and further modulated by ligand substitutions.

In the parent BODIPY **1**, which was only isolated in 2009 [11], we calculated the boron EFG components along x and z to be slightly positive and negative, respectively, which means that obviously here the σ(F) donation “wins” over that of σ(N) (or alternatively, π(N) donation wins over π(F)). Adding methyl groups to the dipyrromethene ligand increases the σ(N) donation, such that the boron EFG component along the N … N direction (*V*_xx_) becomes more negative. As can be seen in Figure 1 and Table 1, this leads to an “inversion” of the boron EFG in compounds **2** and **3** featuring four and five methyl groups, respectively.

In the 14 π‐electron boron bis(oxazol)methanides **4**–**6**, π(N) has more donating character than in the dipyrromethenes (12 π‐electrons), while σ(N) donation is reduced because of the electronegative oxygen atoms. The result is an increased EFG with a larger negative component along the “π(N)” direction (*V*_zz_) and a larger positive component in the N … N direction (*V*_xx_) in the parent **4** relative to **1**. While simple benzo‐fusion of the oxazoles (**5**) has a minor impact, methyl substitution at the benzo rings (**6**) changes the EFG in the same sense as for the BODIPY compounds. It is also interesting to note that the boron EFG in a molecule [10] with the same ligand as in **6** but a BH_2_ instead of a BF_2_ group has the same shape and sign as in **6**, but more than 10 times larger magnitude, which fits the picture of H being an excellent σ but no π donor.

### 2.3 ^11^B‐^19^F Dipole–Dipole Coupling

The dipolar coupling tensor of an ^11^B bonded to a ^19^F spin can be expressed in the “bond” coordinate system by [5, 16]

$$D=\frac{\mu_{0}}{4\pi }\frac{\hbar \gamma_{B}\gamma_{F}}{\pi r^{3}}\left(\begin{array}{ccc} 1/2 & 0 & 0\\ 0 & 1/2 & 0\\ 0 & 0 & -1 \end{array}\right)=D_{\max}\left(\begin{array}{ccc} 1/2 & 0 & 0\\ 0 & 1/2 & 0\\ 0 & 0 & -1 \end{array}\right)$$

(*μ*_0_ = permeability constant, *ħ* = reduced Planck constant, γ_B_ and γ_F_ = gyromagnetic ratios of ^11^B and ^19^F, respectively, and *r* = BF bond length). *D*_max_ is the dipolar coupling constant (= 27.0 kHz for *r* = 1.39 Å) and corresponds to the maximum total splitting in the case the BF bond is along the magnetic field. Similarly to quadrupolar coupling, dipolar coupling leads to a (orientation‐dependent) frequency shift of the ^11^B resonance of –*m*_F_ **D** (*m*_F_ = ^19^F spin state). We can now calculate the combined ^11^B frequency shift **Δν**_**D**_ caused by the two fluorine atoms with the spin states *m*_F1_ and *m*_F2_ by rotating their dipolar coupling tensors into our molecular coordinate system (*θ*= FBF angle bisector):

$${\Delta \nu }_{D}=\frac{D_{\max}}{2}\times$$

$$\begin{array}{c} \left(\begin{array}{ccc} -m_{F1}-m_{F2} & 0 & 0\\ 0 & \left(3\cos^{2}\theta -1\right)\left(m_{F1}+m_{F2}\right) & 3\cos\theta \sin\theta \left(m_{F1}-m_{F2}\right)\\ 0 & 3\cos\theta \sin\theta \left(m_{F1}-m_{F2}\right) & \left(2-3\cos^{2}\theta \right)\left(m_{F1}+m_{F2}\right) \end{array}\right) \end{array}$$

For tetrahedral FBF, *θ* is the magic angle, and **Δν**_**D**_ reduces to

$$∆\nu_{D}=\frac{D_{\max}}{2}\left(\begin{array}{ccc} -m_{F1}-m_{F2} & 0 & 0\\ 0 & 0 & \sqrt{2}\left(m_{F1}-m_{F2}\right)\\ 0 & \sqrt{2}\left(m_{F1}-m_{F2}\right) & m_{F1}+m_{F2} \end{array}\right)$$

The result is a fully rhombic interaction tensor that, for like ^19^F spin states, becomes diagonal in the molecular coordinate system with maxima along x and z, while for unlike spin states it is tilted with maxima at the yz diagonal, reminiscent of a *d*_yz_ orbital. Hence, due to the similar spatial dependence of **Δν**_**Q**_ and **Δν**_**D**_ for like ^19^F spin states, we expect substantial cross‐correlation effects visible as differential linewidths or relaxation times in ^19^F‐coupled ^11^B triplets, provided **Δν**_**Q**_ is not too large. Notably, **Δν**_**D**_ is much less sensitive to the geometry at boron than **Δν**_**Q**_ and within **1**–**6** deviates by less than 5% in any direction. As **Δν**_**Q**_ is opposite for the two ^11^B transitions *m*_B_ = (−1/2 → –3/2) and (+3/2 → +1/2), the effect will only become visible in partially oriented samples, where it will be opposite for the two outer parts of the ^11^B residual quadrupolar triplet.

### 2.4 Partially Oriented Samples

**FIGURE 2:**
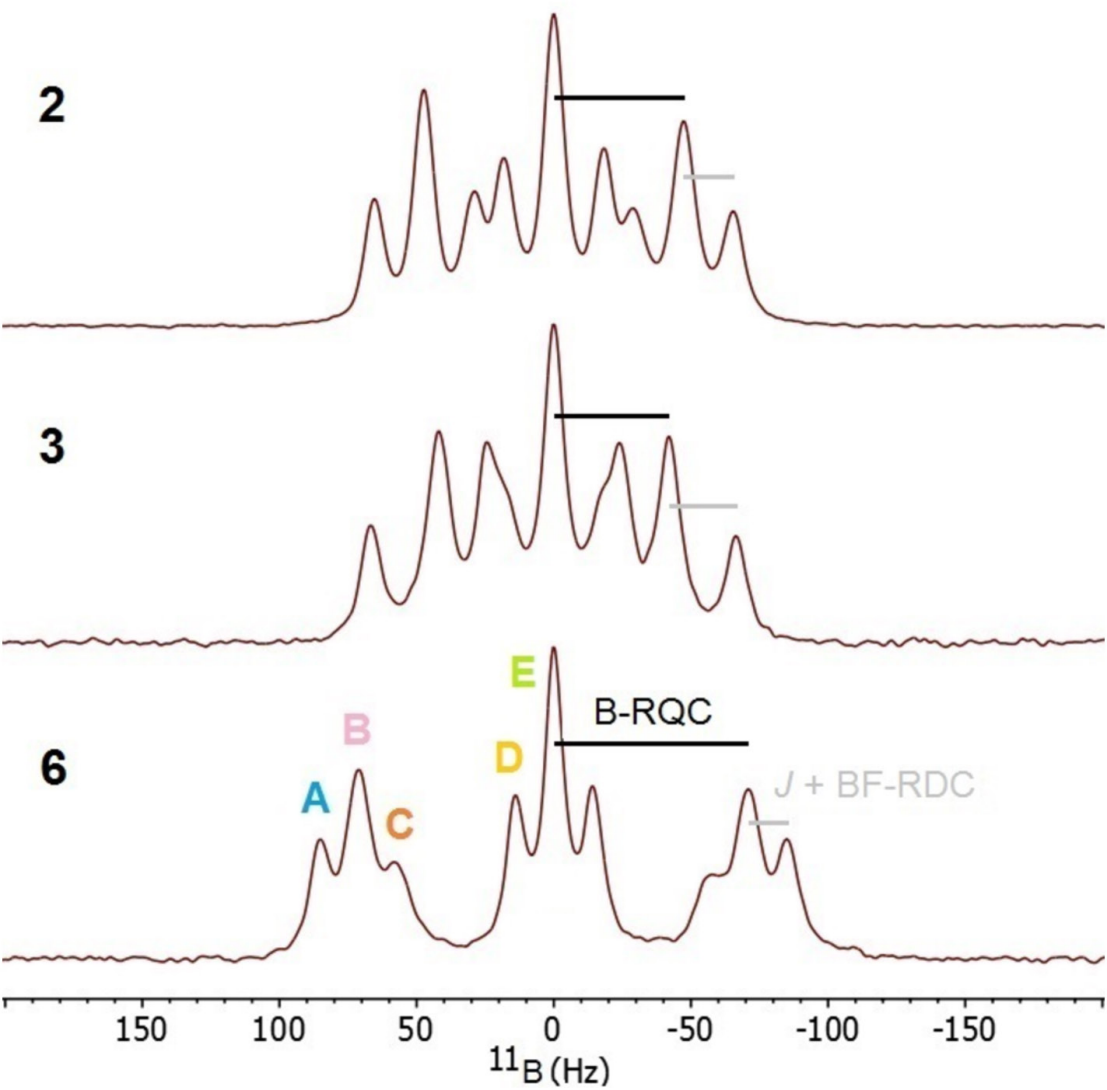
Slice‐selective ^11^B NMR spectra of **2**, **3**, and **6** in PS/THF‐*d*_8_ after 2 weeks of swelling, with ^11^B residual quadrupolar and ^11^B‐^19^F scalar/residual dipolar splittings and selected transitions A–E indicated.

Samples were oriented with crosslinked polystyrene (PS) that was directly swollen in a THF‐*d*_8_ solution of the respective compound [17]. The parent compound **1** is unstable, and **4** and **5** are unknown, so we selected the commercially available compounds **2** and **3**, and **6** that were synthesized recently in our laboratory [10]. After 1 week of swelling, the gels were already homogenous and ready for recording of anisotropic NMR spectra, with only minor changes upon prolonged swelling times (Figures S7, S19, and S33). Figure 2 shows the ^11^B spectra recorded after 15 days.

All three spectra show splitting into a triplet due to RQC (RQC = anisotropic average of **Δν**_**Q**_) that is further split into a triplet due to scalar (*J*) and residual dipolar coupling (RDC = anisotropic average of **Δν**_**D**_) to ^19^F. This is best visible in the spectrum of **6**, while in **2** and **3**—depending on the swelling times—partial overlap occurs because of a splitting ratio close to 2:1. *J* is determined from isotropic solution samples, and the signs of both *J* and the ^11^B‐^19^F RDC are obtained from MSpin fitting [18], the latter along with one‐bond ^13^C‐^1^H RDCs to single alignment tensors (**A**), giving excellent low *Cornilescu* quality factors [19] of 0.021, 0.041, and 0.048 for negative *J* and positive ^11^B‐^19^F RDC (Table 2, Figures S40–S42). **A** is qualitatively similar for **2**, **3**, and **6**, with positive and negative alignment along x and z, respectively.

**TABLE 2:** Experimental couplings and alignment parameters in **2**, **3,** and **6**.

Parameter	2	3	6
*J* (^11^B‐^19^F) (Hz)	−32.6	−32.5	−27.1
^11^B‐^19^F RDC (Hz)	12.8	7.0	11.3
^11^B RQC^exp^ (Hz)	−41.6	−37.6	63.0
^11^B RQC^calc^ (Hz)	−40.5	−37.9	50.3
*A*_xx_ (10^−4^)^a^	6.4	2.9	2.8
*A*_yy_ (10^−4^)^a^	−1.7	−0.9	1.8
*A*_zz_ (10^−4^)^a^	−3.7	−2.0	−4.6

^a^ Obtained from one‐bond ^1^H‐^13^C and ^11^B‐^19^F RDCs.

We obtained the signs of the ^11^B RQCs experimentally from the tilt in ^19^F,^11^B HMQC experiments with small flip angle [7b], which is notably opposite in **6**, confirming an opposite boron EFG relative to **2** and **3** (Figures S12, S24, and S39). We also back‐calculated the ^11^B RQCs from the EFGs and **A**: while for **2** and **3** these are in excellent agreement with the experimental values, the calculated ^11^B RQC in **6** deviates somewhat, indicating the “true” EFG in **6** is underestimated in the DFT calculation by ~20%. Possibly, interactions between the BF_2_ and methyl groups (*J*_CF_ = 7 Hz, Figure S26) have impacts on the F donor strength and FBF angle that are not correctly represented by the DFT model.

### 2.5 ^11^B Quadrupole/^11^B‐^19^F Dipole–Dipole Cross‐Correlation

**FIGURE 3:**
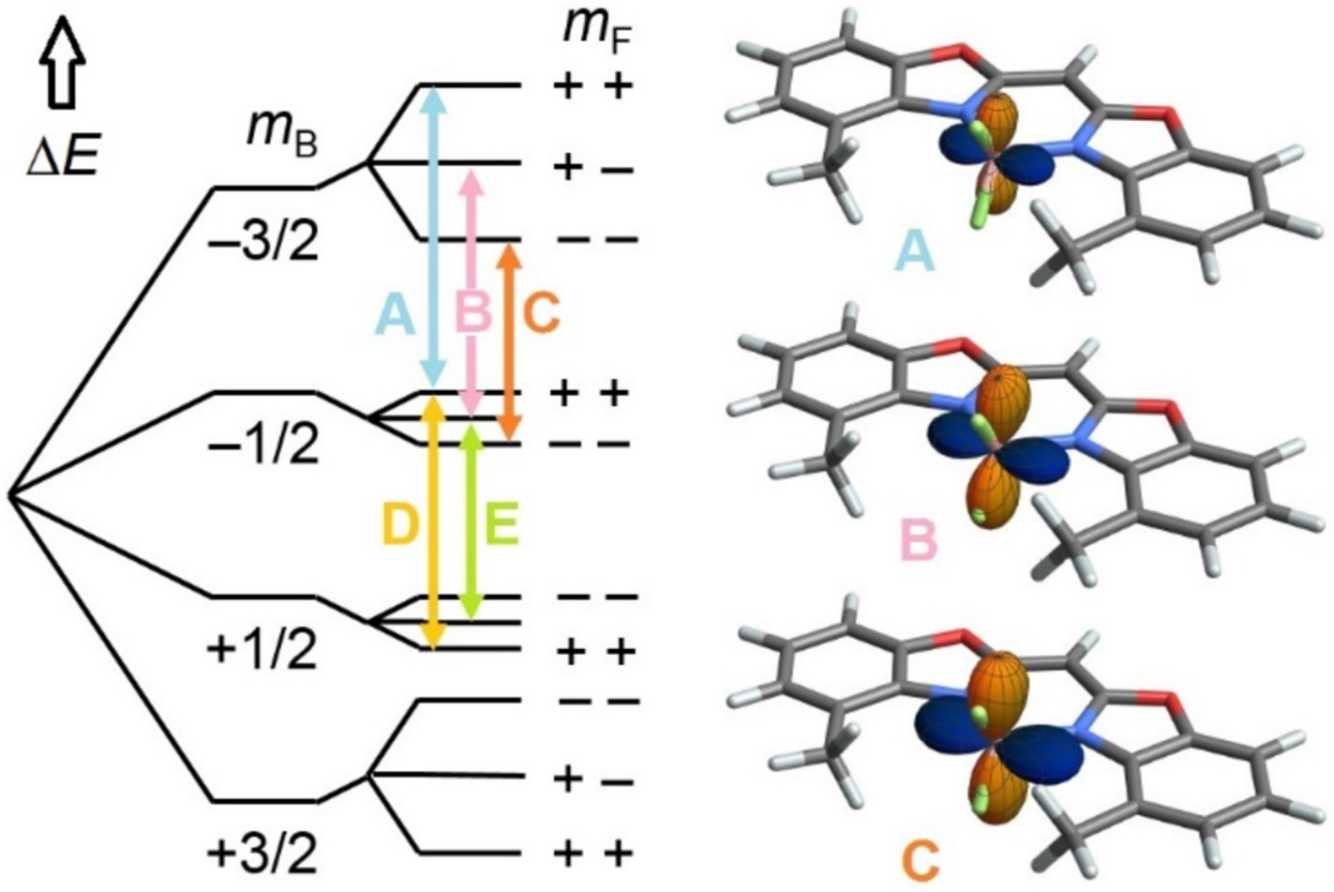
**Left:** ^11^B energy level diagram of weakly aligned **6** with Zeeman splitting followed by quadrupole coupling (^11^B RQC > 0) and scalar/dipolar coupling to ^19^F (*J* + ^11^B,^19^F‐RDC < 0) (from left to right, coupling to ^19^F is strongly exaggerated). ^19^F spin states *m*_F1_ and *m*_F2_ are abbreviated with their signs, and transitions A–E of Figure 2 are highlighted. **Right:** Graphical representations of **Δν**_**Q**_ + **Δν**_**D**_ calculated for transitions A–C (Figure 2) in **6**. Blue and orange represent directions of positive and negative **Δν**_**Q**_ + **Δν**_**D**_, respectively.

As we know the signs of all splittings, we can assign lines A–E of **6** in Figure 2 to transitions in an energy diagram that contains the correct absolute signs of all spin states (Figure 3 **left**). From the calculated EFGs and geometries, we can then calculate the sum **Δν**_**Q**_ + **Δν**_**D**_ for transitions A–C (Figure 3 **right**; values are given in the Supporting Information page S28). The three sum tensors look very similar but differ in their magnitude in a ratio of ~4:5:6, corresponding to destructive (small **Δν**_**Q**_ + **Δν**_**D**_) and constructive (large **Δν**_**Q**_ + **Δν**_**D**_) interference for transitions A and C, respectively. For transition B, the negative region of **Δν**_**Q**_ + **Δν**_**D**_ is somewhat tilted away from the z axis towards the molecular plane.

In the ^11^B spectrum of **6** (Figure 2), the scalar/residual dipolar triplets on either side of the residual quadrupolar triplet are clearly asymmetric, and lineshape fitting gave linewidths of 8.4, 11.0, and 13.1 Hz for transitions A, B, and C, respectively (D = E = 8.4 Hz, Figure S33). This could principally arise from inhomogenous stretching because the quadrupolar triplets expand but the scalar/dipolar triplets contract upon stretching. However, the effect persists in all samples during prolonged swelling and in slice‐selective ^11^B spectra of 1.3 mm thin slices [20]. Additionally, slice‐selective spectra recorded 0.5 mm above, 0.5 mm below, and in the center of the coil show virtually identical splittings (Figure S34), so that we can safely neglect sample inhomogeneity.

The most likely explanation for the observed effect is ^11^B quadrupole/^11^B‐^19^F dipole–dipole cross‐correlation, that is, constructive or destructive interference of **Δν**_**Q**_ and **Δν**_**D**_ that leads to larger or smaller field modulations at the ^11^B nucleus as the molecule tumbles. The ^19^F spin states are relatively long‐lived (*T*_1_ ~0.5 s, Table S7), such that no interference from ^19^F relaxation is expected. Due to partial overlap, linewidths in **2** and **3** are less reliable, but the effect seems to be less pronounced (8.7 vs. 9.6 Hz and 8.8 vs. 12.4 Hz for the corresponding transitions A vs. C in **2** and **3**, respectively, Figures S7 and S19). Here, the order of the satellite transitions *m*_B_ = (−1/2 → –3/2) and (+3/2 → +1/2) of the ^11^B quadrupolar triplet is reversed along with the boron EFG so that the lines should be affected in a similar manner as in **6**.

## 3 Discussion

We have not yet attempted to relate our measured linewidths to the calculated magnitudes of |**Δν**_**Q**_ + **Δν**_**D**_| or |**Δν**_**Q**_||**Δν**_**Q**_| of the individual transitions. Intuitively, we would expect a quadratic dependence on |**Δν**_**Q**_ + **Δν**_**D**_|, but relaxation in a spin‐3/2 system is more complex as the three single‐quantum transitions may not be regarded separately. This becomes clear, for example, from the measured linewidths of transitions D–E that are not directly affected by the first‐order quadrupole interaction yet are comparable to transitions A–C. In fact, all three single‐quantum transitions in a spin‐3/2 nucleus ideally have identical linewidths (i.e., they do not depend on individual interaction tensors) under fast tumbling conditions until they start to diverge at *ω*_0_τ_C_ ~1 (*ω*_0_ = ^11^B Larmor frequency, τ_C_ = tumbling time) [15, 21]. This could also explain why in our case differential line‐broadening within the satellite transitions A–C was less pronounced in the relatively “lighter” molecules **2** and **3** compared with “heavier” **6** that is closer to *ω*_0_τ_C_ = 1.

It has been claimed in literature that quadrupole/dipole–dipole cross correlation only leads to differential longitudinal relaxation and not to differential line broadening [5, 22]. However, to the best of our knowledge, this has not been theoretically fully treated for the general case of rhombic EFGs [5], and experimental support is yet sparse, including relaxation of ^2^H in an intermolecular dipolar field from paramagnetic ions in the alignment medium and (differential) relaxation of spin‐1/2 multiplets scalar/dipolar coupled to quadrupolar nuclei [22]. Both situations are quite far from ours.

We have measured ^11^B inversion recovery *T*_1_ relaxation times of our anisotropic samples of **2**, **3**, and **6**, and found uniform values for all multiplet lines (Table S7). This is in line with the report of CHDCl_2_ in the liquid crystalline phase, where unselective inversion gave uniform, monoexponential relaxation behavior due to spin‐mixing, while selective inversion was needed to stimulate cross‐correlation effects [6]. In our case, the multiplet lines are too close to achieve clean selective inversion. We have also not attempted to determine *T*_2_ relaxation times by CPMG‐type experiments, as these would likewise be affected by spin‐mixing.

Notably, we have also calculated ^11^B chemical shielding anisotropy in **6** and found that it is also highly rhombic, with smallest and largest shielding along x and z, respectively (Δσ_zz_ ~ −Δσ_xx_ ~ 7 ppm). This would principally make cross‐correlation with the other interactions visible as asymmetric distortions of the quadrupolar and scalar/dipolar triplets. However, this effect will be small as even at our highest field (*B*_0_ = 14.4 T), the associated frequency shifts (**Δν**_**S**_ = –γ_B_ *B*_0_ **Δσ**/2π) are ~10 times smaller than **Δν**_**D**_ and ~50 times smaller than **Δν**_**Q**_.

Finally, we have started searching for compounds in which the EFG is even smaller, so that more pronounced cross‐correlation effect would be expected than in **1**–**6**. These include BODIPY dyes **7**–**9** with fewer methyl groups and a bis(benzoxazol)methanide with more bulky isopropyl substituents **10** (Figure S43, Tables S8 and S9). These molecules could be synthesized and studied at lower temperatures in order to shift the single‐quantum transitions closer to *ω*_0_τ_C_ = 1. The ultimate goal of our work is to derive proper equations and to obtain a deeper understanding of quadrupole/dipole–dipole cross correlation.

## Supporting information


**Figure S1.** 400.3 MHz ^1^H NMR spectrum of compound **2** in THF‐*d*8. Residual solvent signals are marked with #.
**Figure S2.** 100.7 MHz ^13^C{^1^H} NMR spectrum of compound **2** in THF‐*d*8. Residual solvent signals are marked with #. The methyl groups close to the BF_2_ group show a splitting of *J*C‐F = 2.1 Hz.
**Figure S3.** 128.4 MHz ^11^B NMR spectrum of compound **2** in THF‐*d*8. The splitting corresponds to ^1^ *J*B‐F = −32.6 Hz.
**Figure S4.** 376.6 MHz ^19^F NMR spectrum of compound **2** in THF‐*d*8. The splitting corresponds to ^1^ *J*B‐F = −32.6 Hz.
**Figure S5.** 400.3/100.7 MHz ^1^H,^13^C CLIP‐HSQC spectrum of compound **2** in THF‐*d*8. Residual solvent signals are marked #.
**Figure S6.** 160.5 MHz ^11^B NMR spectrum of compound **2** in PS/THF‐*d*8 after 7 days of swelling. The splittings correspond to ^1^ *T*B‐F = −19.8 Hz and ^11^B RQC = −41.6 Hz.
**Figure S7.** 128.4 MHz ^11^B NMR spectrum of a 1.3 mm slice at the center of the NMR coil of compound **2** in PS/THF‐*d*8 after 15 days of swelling with a lineshape fitting performed on the multiplet.
**Table S1.** Lineshape fitting parameters for the ^11^B multiplet of compound **2** as shown in **Figure S7**. The L/G parameter was fixed to 0.75 for all signals. Widths include LB = 2 Hz from exponential multiplication.
**Figure S8.** 160.5 MHz ^11^B{^19^F} NMR spectrum of compound **2** in PS/THF‐*d*8 after 7 days of swelling. The splitting corresponds to ^11^B RQC = −41.6 Hz.
**Figure S9.** 470.7 MHz ^19^F NMR spectrum of compound **2** in PS/THF‐*d*8 after 7 days of swelling.
**Figure S10.** 470.7 MHz ^19^F{^11^B} NMR spectrum of compound **2** in PS/THF‐*d*8 after 7 days of swelling. The splitting corresponds to ^2^ *D*F‐F = 12.1 Hz. The broad shoulder on the left side of the signal belongs to the ^10^B isotopologue.
**Figure S11.** 500.3/125.8 MHz ^1^H,^13^C CLIP‐HSQC spectrum of compound **2** in PS/THF‐*d*8 after 7 days of swelling. Residual solvent signals are marked with #. Signals marked with * belong to unpolymerized styrene. The broad signals between δ(^13^C) = 40–50 ppm and 125–130 ppm are from polystyrene. The 1D traces (^1^H and ^13^C) were taken from the isotropic spectra.
**Figure S12.** 470.7/160.5 MHz ^19^F,^11^B HMQC spectrum of compound **2** in PS/THF‐*d*8 after 7 days of swelling with a ^11^B flip angle of 30°. From the tilt of the signal, a negative ^11^B RQC can be deduced.
**Figure S13.** 400.3 MHz ^1^H NMR spectrum of compound **3** in THF‐*d*8. Residual solvent = #, trace water = *.
**Figure S14.** 100.7 ^13^C{^1^H} NMR spectrum of compound **3** in THF‐*d*8. Residual solvent signals are marked with #. The methyl groups close to the BF_2_ group show a splitting of *J*C‐F = 2.1 Hz.
**Figure S15.** 128.4 MHz ^11^B NMR spectrum of compound **3** in THF‐*d*8. The splitting corresponds to ^1^ *J*B‐F = −32.5 Hz.
**Figure S16.** 376.6 MHz ^19^F NMR spectrum of compound **3** in THF‐*d*8. The splitting corresponds to ^1^ *J*B‐F = −32.5 Hz.
**Figure S17.** 400.3/100.7 MHz ^1^H,^13^C CLIP‐HSQC spectrum of compound **3** in THF‐*d*8. Residual solvent signals are marked #.
**Figure S18.** 160.5 MHz ^11^B NMR spectrum of compound **3** in PS/THF‐*d*8 after 7 days of swelling. The splittings correspond to ^1^ *T*B‐F = −25.5 Hz and ^11^B RQC = −37.6 Hz.
**Figure S19.** 128.4 MHz ^11^B NMR spectrum of a 1.3 mm slice at the center of the NMR coil of compound **3** in PS/THF‐*d*8 after 15 days of swelling with a lineshape fitting performed on the multiplet.
**Table S2.** Lineshape fitting parameters for the ^11^B multiplet of compound **3** as shown in **Figure S19**. The L/G parameter was fixed to 0.75 for all signals. Widths include LB = 2 Hz from exponential multiplication.
**Figure S20.** 160.5 MHz ^11^B{^19^F} NMR spectrum of compound **3** in PS/THF‐*d*8 after 7 days of swelling. The splitting corresponds to ^11^B RQC = −37.6 Hz.
**Figure S21.** 470.7 MHz ^19^F NMR spectrum of compound **3** in PS/THF‐*d*8 after 7 days of swelling.
**Figure S22.** 470.7 MHz ^19^F{^11^B} NMR spectrum of compound **3** in PS/THF‐*d*8 after 7 days of swelling. The splitting corresponds to ^2^ *D*F‐F = 7.1 Hz. The broad shoulder on the left side of the signal belongs to the ^10^B isotopologue.
**Figure S23.** 500.3/125.8 MHz ^1^H,^13^C CLIP‐HSQC spectrum of compound **3** in PS/THF‐*d*8 after 7 days of swelling. Residual solvent signals are marked with #. The broad signals between δ(^13^C) = 40–50 ppm and 125–130 ppm are of polystyrene. The 1D traces (^1^H and ^13^C) were taken from the isotropic spectra.
**Figure S24.** 470.7/160.5 MHz ^19^F,^11^B HMQC spectrum of compound **3** in PS/THF‐*d*8 after 7 days of swelling with a ^11^B flip angle of 30°. From the tilt of the signal, a negative ^11^B RQC can be deduced.
**Figure S25.** 600.3 MHz ^1^H NMR spectrum of compound **6** in THF‐*d*8. The residual solvent signals are marked with #. The signals marked with * stem from residual ligand and diethyl ether.
**Figure S26.** 125.8 MHz ^13^C{^1^H} NMR spectrum of compound **6** in THF‐*d*8. Residual solvent signals are marked with #. The signals marked with * stem from residual ligand. The methyl groups show a splitting of *J*C‐F = 7.0 Hz.
**Figure S27.** 160.5 MHz 11B NMR spectrum of compound **6** in THF‐*d*8. The signals marked with * belong to impurities (BF_4_^−^ and BF_3_·OEt_2_). The splitting corresponds to (1*J*B‐F = −27.1 Hz).
**Figure S28.** 160.5 MHz ^11^B{^19^F} NMR spectrum of compound **6** in THF‐*d*8. The signals marked with * belong to impurities (BF_4_^−^ and BF_3_·OEt_2_).
**Figure S29.** 470.7 MHz 19F NMR spectrum of compound **6** in THF‐*d*8. The signals marked with * belong to impurities (BF_3_·OEt_2_ and BF_4_^−^). The splitting corresponds to ^1^ *J*B‐F = −27.1 Hz.
**Figure S30.** 564.7 MHz ^19^F{^11^B} NMR spectrum of compound **6** in THF‐*d*8. The signals marked with * belong to impurities (BF_3_·OEt_2_ and BF_4_^−^). The broad shoulder on the left side of the signal belongs to the ^10^B isotopologue.
**Figure S31.** 400.3/100.7 MHz ^1^H,^13^C CLIP‐HSQC spectrum of compound **6** in THF‐*d*8. Residual solvent signals are marked with #. The signals marked with * are from impurities.
**Figure S32.** 160.5 MHz ^11^B NMR spectrum of compound **6** in PS/THF‐*d*8 after 7 days of swelling. The signals marked with * are from impurities (BF_4_^−^ and BF_3_·OEt_2_). The splittings correspond to ^1^ *T*B‐F = −15.8 Hz and ^11^B RQC = 63.0 Hz.
**Figure S33.** 128.4 MHz ^11^B NMR spectrum of a 1.3 mm slice at the center of the NMR coil of compound **6** in PS/THF‐*d*8 after 15 days of swelling with a lineshape fitting performed on the multiplet.
**Table S3.** Lineshape fitting parameters for the ^11^B multiplet of compound **6** as shown in **Figure S33**. The L/G parameter was fixed to 0.75 for all signals. Widths include LB = 2 Hz from exponential multiplication.
**Figure S34.** 128.4 MHz 1.3 mm slice‐selective ^11^B NMR spectra of compound **6** in PS/THF‐*d*8 after 15 days of swelling. The spectra were recorded at positions + 0.5 mm (top), 0 mm (center), and −0.5 mm relative to the center of the coil.
**Figure S35.** 160.5 MHz ^11^B{^19^F} NMR spectrum of compound **6** in PS/THF‐*d*8 after 7 days of swelling. The signals marked with * are impurities (BF_4_^−^ and BF_3_·OEt_2_). The splitting corresponds to ^11^B RQC = 63.0 Hz.
**Figure S36.** 470.7 MHz ^19^F NMR spectrum of compound **6** in PS/THF‐*d*8 after 7 days of swelling.
**Figure S37.** 470.7 MHz ^19^F{^11^B} NMR spectrum of compound **6** in PS/THF‐*d*8 after 7 days of swelling. The splitting corresponds to ^2^ *D*F‐F = 18.0 Hz. The broad shoulder on the left side of the signal belongs to the ^10^B isotopologue.
**Figure S38.** 500.3/125.8 MHz ^1^H,^13^C CLIP‐HSQC spectrum of compound **6** in PS/THF‐*d*8 after 7 days of swelling. Residual solvent signals are marked with #. Signals marked with * belong to unpolymerized styrene. The broad signals between δ(^13^C) = 40–50 ppm and 125–130 ppm are from polystyrene. The 1D traces (^1^H and ^13^C) were taken from the isotropic spectra.
**Figure S39.** 470.7/160.5 MHz ^19^F,^11^B HMQC spectrum of compound **6** in PS/THF‐*d*8 after 7 days of swelling with a ^11^B flip angle of 30°. From the tilt of the signal, a positive ^11^B RQC can be deduced.
**Figure S40.** Graphical representation of the alignment tensor (obtained from 5 one‐bond RDCs) for compound **2** in the molecular coordinate system with the positions numbered. Green and red lobes correspond to directions of positive and negative alignment, respectively. Principal values, given in the molecular coordinate system, are *A*xx = 6.388·10^−4^, *A*yy = −1.718·10^−4^, and *A*zz = −3.669·10^−4^. The Cornilescu quality factor^[11]^ is 0.021.
**Table S4.** Experimental chemical shifts (in ppm, determined from the isotropic sample) and one‐bond coupling constants (in Hz, where *T* = *J* + *D*) obtained from a sample of compound **2** in PS/THF‐d8 after 7 days of swelling. The experimental ^11^B RQC is −41.6 Hz, the calculated value is 40.5 Hz. Numbering of the positions is according to **Figure S40**.
**Figure S41.** Graphical representation of the alignment tensor (obtained from 5 one‐bond RDCs) for compound **3** in the molecular coordinate system with the positions numbered for discussion. The green and red lobes correspond to directions of positive and negative alignment, respectively. Principal values, given in the molecular coordinate system, are *A*xx = 2.898·10^−4^, *A*yy = −9.114·10^−5^, and *A*zz = −1.986·10^−4^. The Cornilescu quality factor is 0.041.
**Table S5.** Experimental chemical shifts (in ppm, determined from the isotropic sample) and one‐bond coupling constants (in Hz, where *T* = *J* + *D*) obtained from a sample of compound **3** in PS/THF‐d8 after 7 days of swelling. The experimental ^11^B RQC is −37.6 Hz, the calculated value is −37.9 Hz. Numbering of the positions is according to **Figure S41**.
**Figure S42.** Graphical representation of the alignment tensor (obtained from 5 one‐bond RDCs) for compound **6** in the molecular coordinate system with the positions numbered for discussion. The green and red lobes correspond to directions of positive and negative alignment, respectively. Principal values, given in the molecular coordinate system, are *A*xx = 2.760·10^−4^, *A*yy = 1.793·10^−4^, and *A*zz = −4.553·10^−4^. The Cornilescu quality factor is 0.048.
**Table S6.** Experimental chemical shifts (in ppm, determined from the isotropic sample) and one‐bond coupling constants (in Hz, where *T* = *J* + *D*) obtained from a sample of compound **6** in PS/THF‐d8 after 7 days of swelling. The experimental ^11^B RQC is 63.0 Hz, the calculated value is 50.3 Hz. Numbering of the positions is according to **Figure S42**.
**Table S7.** *T*_1_ relaxation times for ^11^B (128 MHz) and ^19^F (376 MHz) of **2**, **3,** and **6** in PS/THF‐*d*8.
**Figure S43.** Geometry‐optimized structures **7**–**10**. Boron EFG tensors are shown with the same scaling factor as in Figure 1.
**Table S8.** Selected bond lengths (BN, BF) and angles (NBN, FBF) in geometry‐optimized structures **7**–**10**.
**Table S9.** EFG components in geometry‐optimized structures **7**–**10**.
