# Supplementary material for: Cross‐Correlation Between 11B Quadrupole and 11B‐19F Dipole–Dipole Coupling in BF2 Groups
Source: Magn Reson Chem. 2024 Dec 20;63(3):220–6. doi: 10.1002/mrc.5507 (PMC11788097; doi:10.1002/mrc.5507)
Supplement: Supplementary file 1 — Figure S1. 400.3 MHz 1H NMR spectrum of compound 2 in THF‐d8. Residual solvent signals are marked with #. Figure S2. 100.7 MHz 13C{1H} NMR spectrum of compound 2 in THF‐d8. Residual solvent signals are marked with #. The methyl groups close to the BF2 group show a splitting of JC‐F = 2.1 Hz. Figure S3. 128.4 MHz 11B NMR spectrum of compound 2 in THF‐d8. The splitting corresponds to 1 JB‐F = −32.6 Hz. Figure S4. 376.6 MHz 19F NMR spectrum of compound 2 in THF‐d8. The splitting corresponds to 1 JB‐F = −32.6 Hz. Figure S5. 400.3/100.7 MHz 1H,13C CLIP‐HSQC spectrum of compound 2 in THF‐d8. Residual solvent signals are marked #. Figure S6. 160.5 MHz 11B NMR spectrum of compound 2 in PS/THF‐d8 after 7 days of swelling. The splittings correspond to 1 TB‐F = −19.8 Hz and 11B RQC = −41.6 Hz. Figure S7. 128.4 MHz 11B NMR spectrum of a 1.3 mm slice at the center of the NMR coil of compound 2 in PS/THF‐d8 after 15 days of swelling with a lineshape fitting performed on the multiplet. Table S1. Lineshape fitting parameters for the 11B multiplet of compound 2 as shown in Figure S7. The L/G parameter was fixed to 0.75 for all signals. Widths include LB = 2 Hz from exponential multiplication. Figure S8. 160.5 MHz 11B{19F} NMR spectrum of compound 2 in PS/THF‐d8 after 7 days of swelling. The splitting corresponds to 11B RQC = −41.6 Hz. Figure S9. 470.7 MHz 19F NMR spectrum of compound 2 in PS/THF‐d8 after 7 days of swelling. Figure S10. 470.7 MHz 19F{11B} NMR spectrum of compound 2 in PS/THF‐d8 after 7 days of swelling. The splitting corresponds to 2 DF‐F = 12.1 Hz. The broad shoulder on the left side of the signal belongs to the 10B isotopologue. Figure S11. 500.3/125.8 MHz 1H,13C CLIP‐HSQC spectrum of compound 2 in PS/THF‐d8 after 7 days of swelling. Residual solvent signals are marked with #. Signals marked with * belong to unpolymerized styrene. The broad signals between δ(13C) = 40–50 ppm and 125–130 ppm are from polystyrene. The 1D traces (1H and 13C) were taken from the isotro [file MRC-63-220-s001.pdf]

Cross-correlation between ^{11}B quadrupole and ^{11}B-^{19}F dipole-dipole coupling in BF_2 groups

Franziska Rüttger, Dominik Franke, Jannik Probst, Xiaobai Wang, Dietmar Stalke, and Michael John*

Institut für Anorganische Chemie, Tammannstrasse 4, 37077 Göttingen, Germany

Email: mjohn@gwdg.de

Supporting Information

Contents

Preparation of polystyrene sticks S2
NMR sample preparation S2
NMR measurements S2
DFT calculations and other Software S2
NMR spectra of compound **2** S3
NMR spectra of compound **3** S10
NMR spectra of compound **6** S17
Alignment tensors S25
Interference of quadrupolar and dipolar frequency shifts in **6** S28
Relaxation times S28
Further structures S29
References S30

Preparation of polystyrene sticks

Styrene was separated from its radical inhibitor using a dry column packed with silica gel and dried over $MgSO_4$. It was then transferred into a Schlenk flask under argon atmosphere and degassed with three freeze-pump cycles. 0.2 vol% of 1,3-divinylbenzene (DVB) was added while stirring and the mixture was transferred into glass tubes of 3.4 mm inner diameter. The polymerization was performed at 115 °C for three days. The glass tubes were broken and the homogeneous parts of the polystyrene were cut into sticks of ~1.5 cm length. The sticks were stored and handled in an argon dry box.

NMR sample preparation

Compounds **2** and **3** were commercially obtained from BLDpharm (Shanghai, China) and TCI (Tokyo, Japan), respectively, and used without further purification. Compound **6** was synthesized according to reference [1] and stored and handled in an argon dry box. Anisotropic samples of compound **6** were prepared inside an argon dry box. The compound (5-10 mg) was dissolved in 0.5 mL dry THF-d_8 and filled into a standard 5 mm (ID 4.2 mm) quartz NMR tube. The polystyrene stick was inserted into the active coil region of the NMR tube and held horizontally until the stick had swollen up to the walls of the NMR tube. After roughly five minutes, it could be put back into an upright position. Anisotropic samples of compounds **2** and **3** were prepared analogous to that of compound **6** under non-inert conditions (here, compound **3** suffered from very low solubility in THF-d_8). All samples were swollen at room temperature for 7 days for most NMR experiments, and for 15 days for slice-selective ^{11}B NMR experiments.

NMR measurements

NMR measurements were performed on a Bruker Avance III HD 400 MHz spectrometer with BBI probe, a Bruker Avance Neo 400 MHz spectrometer with BBO cryoprobe prodigy, a Bruker Avance III HD 500 MHz spectrometer with BBO cryoprobe prodigy and a Bruker Avance Neo 600 MHz spectrometer with TBO probe, all with z-gradients. All measurements were performed at 298 K. Routine one-dimensional spectra were recorded with high power (~25 kHz) 30° flip angle and optional Waltz-16 (^{1}H) or GARP (^{11}B, ^{19}F) decoupling with decoupling strengths of ~2.5 kHz. ^{13}C-^{1}H coupling constants were measured from CLIP-HSQC spectra.[2] Slice-selective ^{11}B NMR spectra were recorded as described in reference [3] with a gradient strength of 53 G/cm and a 1 ms G4 excitation pulse (corresponding to a slice of 1.3 mm) and an offset of 7.24 kHz/mm. ^{19}F,^{11}B HMQC spectra were recorded with the cnst2 parameter set to $^{1}T(^{19}F,^{11}B)$ and a ^{11}B flip angle of 30° as introduced in reference [4]. ^{11}B and ^{19}F T_1 relaxation times were measured using inversion recovery with 8 different delays at 128 and 376 MHz, respectively. Processing and analysis of NMR data was performed with TopSpin 4.0.8 and MestReNova 14.1.2.

DFT calculations and other Software

Determination and graphical representation of alignment tensors was performed using the software MSpin.[5] DFT calculations were performed with Gaussian16[6] at the B3LYP/Def2TZVP[7]/GD3BJ[8] level of theory. Starting geometries and graphical representations were created with Avogadro.[9] Graphical representations of EFG tensors were created with a notebook by J. Autschbach with Mathematica 13.1 (Wolfram Research Inc. Champaign, Illinois, USA).[10]

NMR spectra of compound **2**

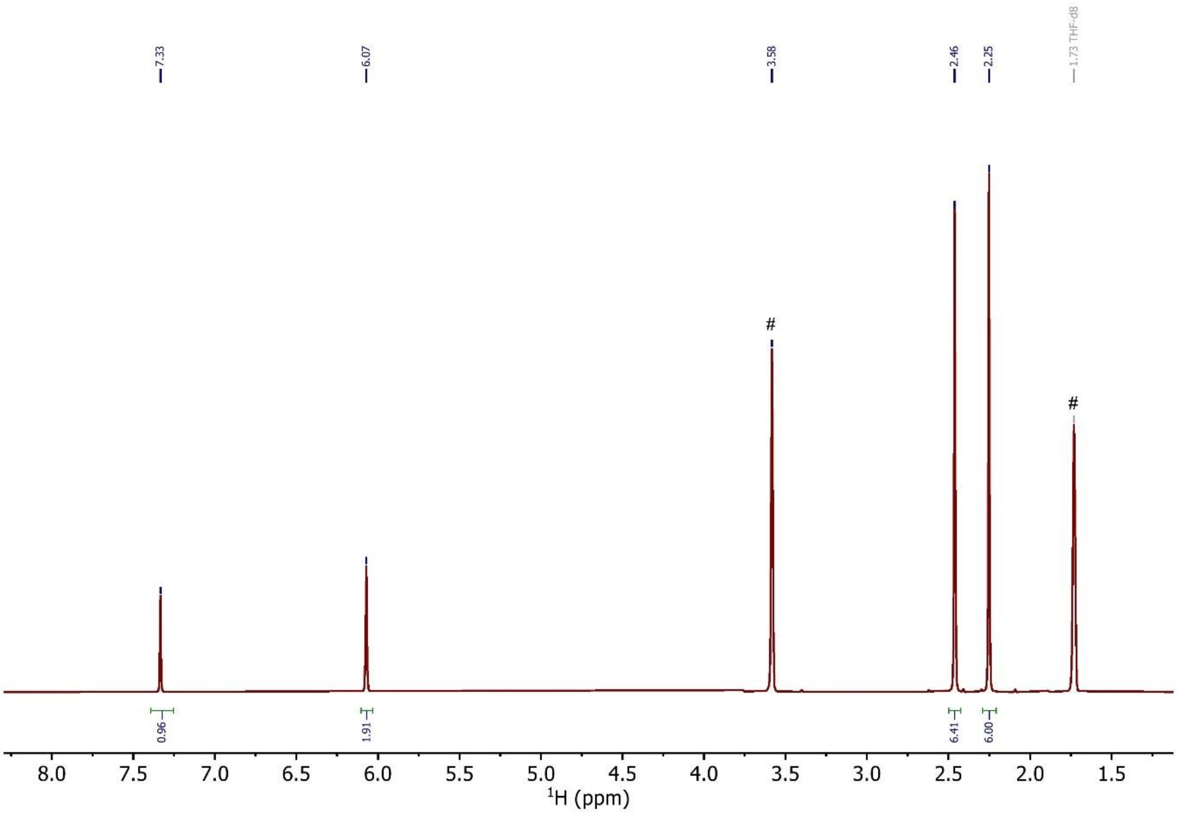

Figure S1. 400.3 MHz ^{1}H NMR spectrum of compound **2** in THF-d_8. Residual solvent signals are marked with #.

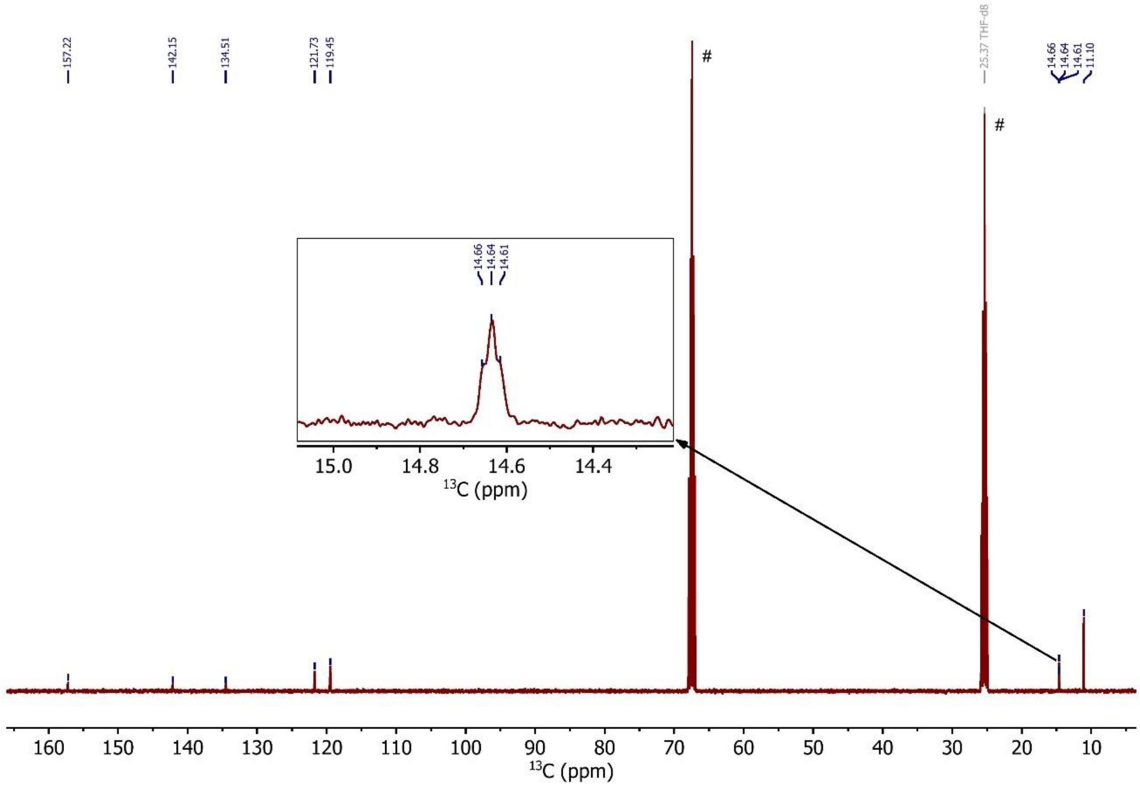

Figure S2. 100.7 MHz $^{13}C\{^{1}H\}$ NMR spectrum of compound **2** in THF-d_8. Residual solvent signals are marked with #. The methyl groups close to the BF_2 group show a splitting of $J_{C\text{-}F}$ = 2.1 Hz.

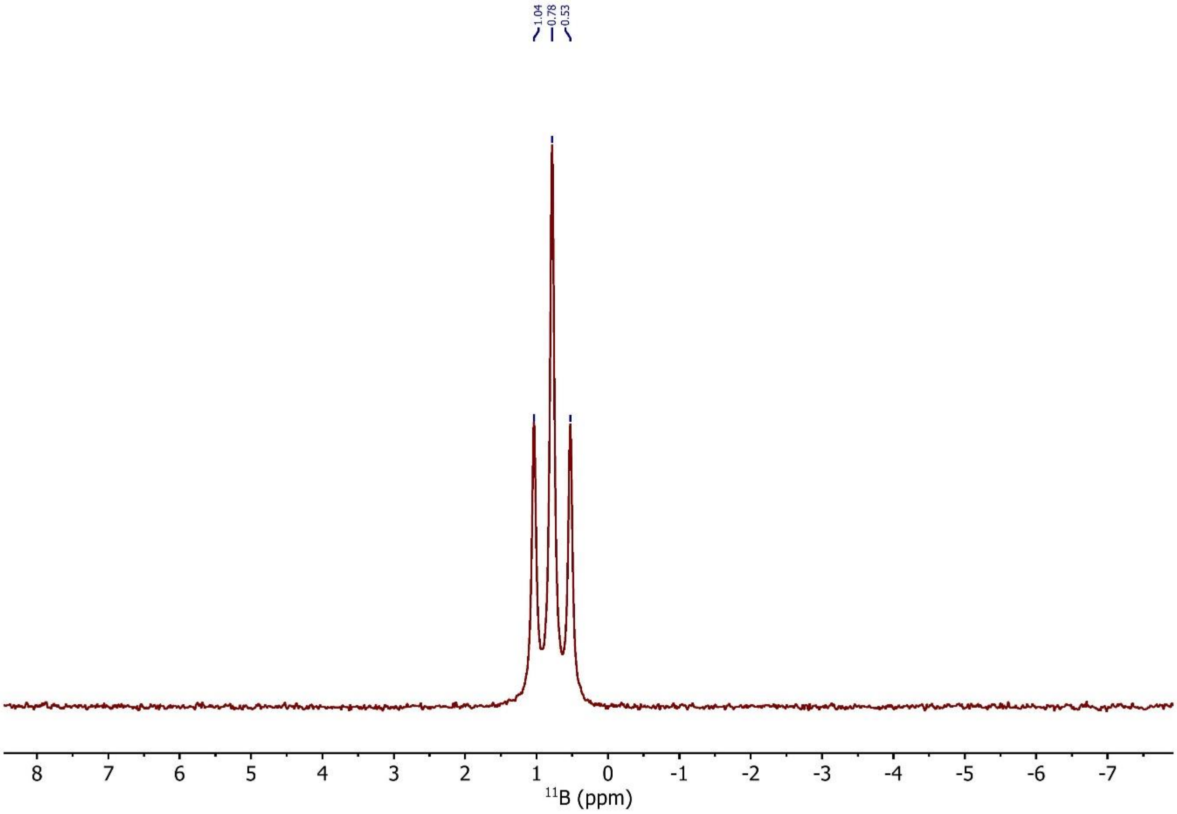

Figure S3. 128.4 MHz ^{11}B NMR spectrum of compound **2** in THF-d_8. The splitting corresponds to $^{1}J_{B\text{-}F} = -32.6$ Hz.

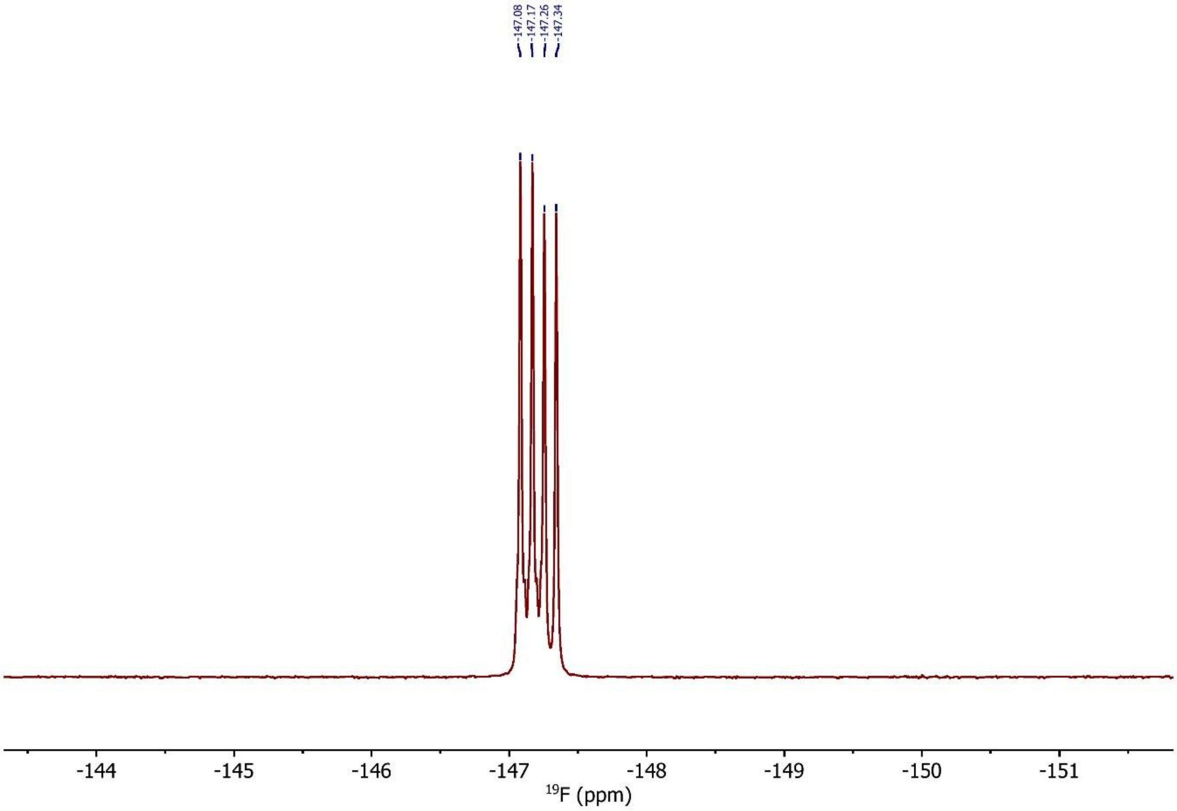

Figure S4. 376.6 MHz ^{19}F NMR spectrum of compound **2** in THF-d_8. The splitting corresponds to $^{1}J_{B\text{-}F} = -32.6$ Hz.

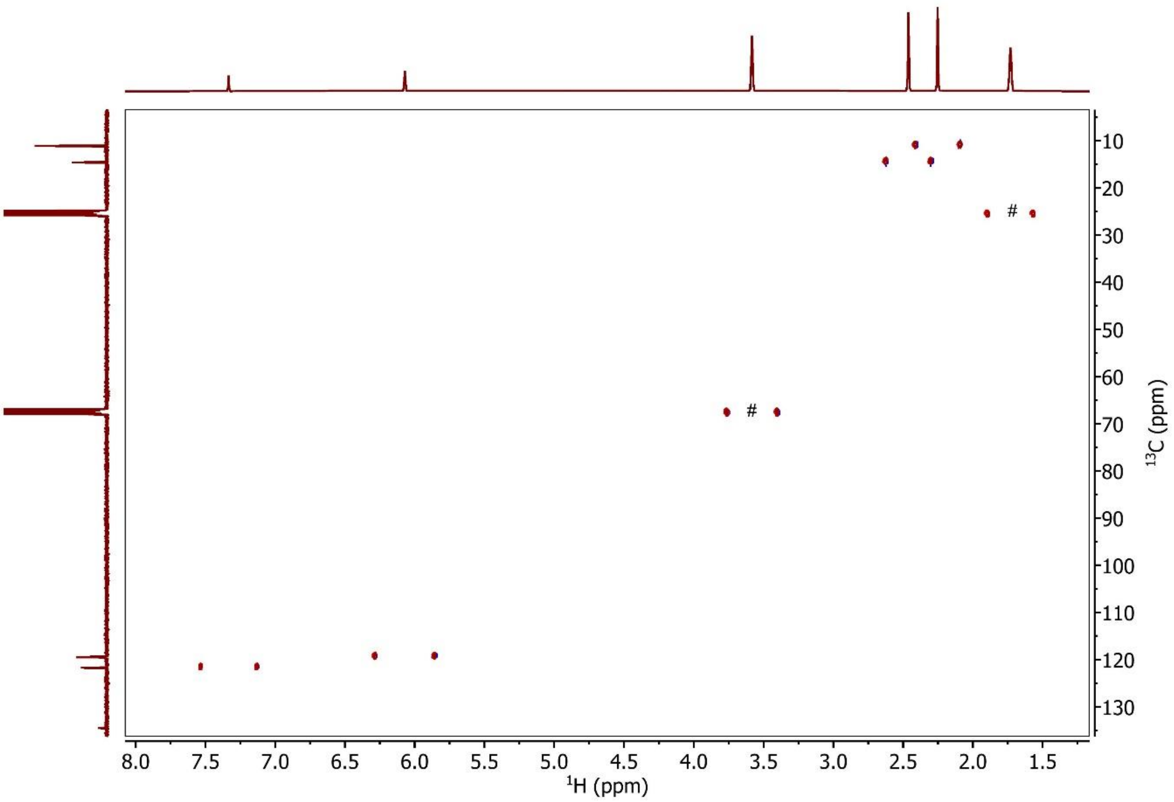

Figure S5. 400.3/100.7 MHz $^{1}H,^{13}C$ CLIP-HSQC spectrum of compound **2** in THF-d_8. Residual solvent signals are marked #.

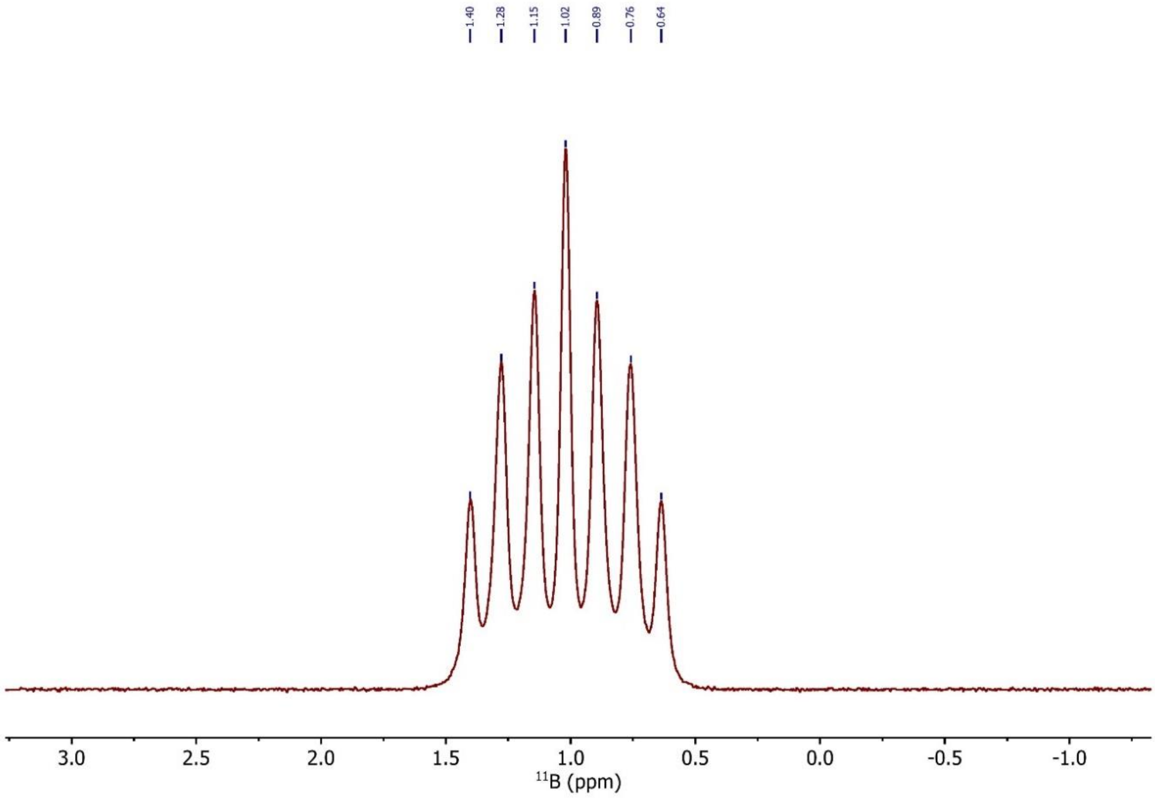

Figure S6. 160.5 MHz ^{11}B NMR spectrum of compound **2** in PS/THF-d_8 after seven days of swelling. The splittings correspond to $^{1}T_{B\text{-}F}$ = –19.8 Hz and ^{11}B RQC = –41.6 Hz.

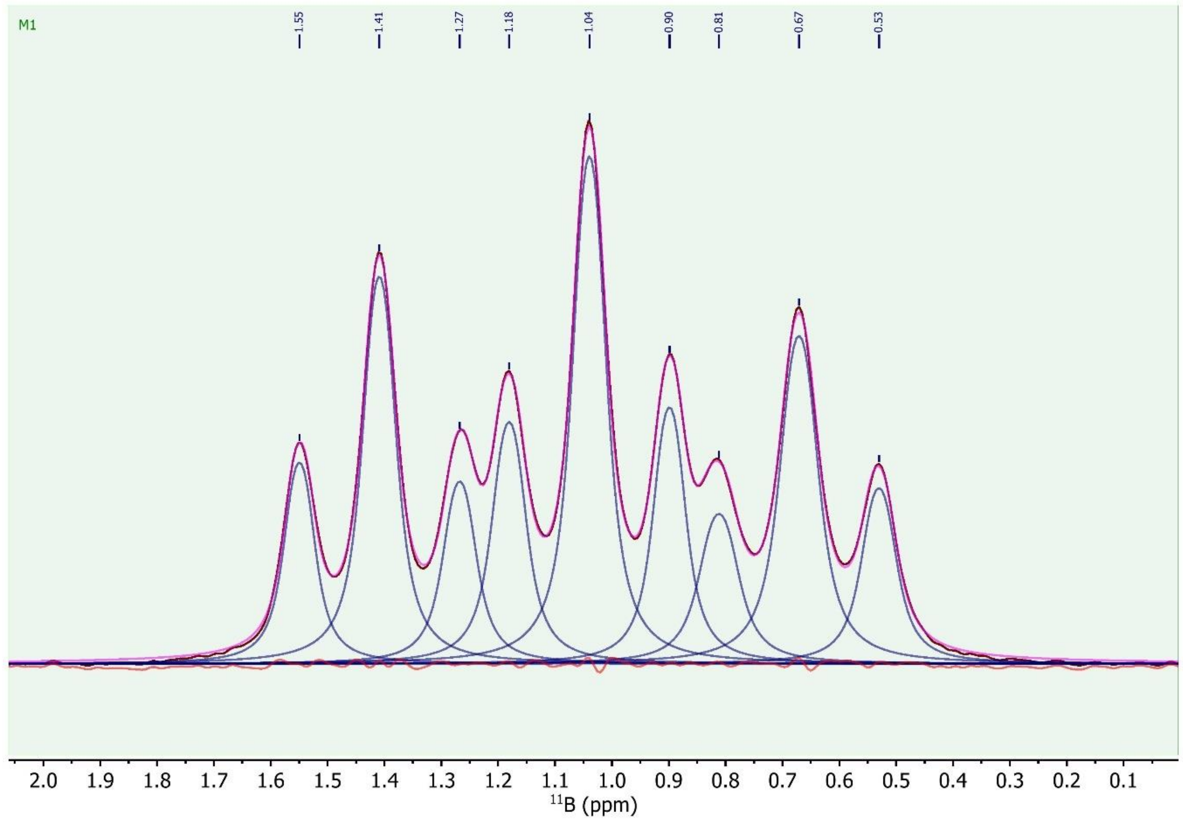

Figure S7. 128.4 MHz ^{11}B NMR spectrum of a 1.3 mm slice at the center of the NMR coil of compound **2** in PS/THF-d_8 after fifteen days of swelling with a lineshape fitting performed on the multiplet.

Table S1. Lineshape fitting parameters for the ^{11}B multiplet of compound **2** as shown in **Figure S7**. The L/G parameter was fixed to 0.75 for all signals. Widths include LB = 2 Hz from exponential multiplication.

δ (ppm)	Height	Width (Hz)	Area
1.550	1378537	8.2	24746319
1.409	2651342	8.8	51324144
1.268	1248888	8.8	24229857
1.181	1655384	9.0	32897755
1.040	3474082	8.9	67763481
0.899	1756396	8.6	33074697
0.812	1026231	10.4	23545595
0.671	2244824	10.1	49989072
0.530	1202452	9.2	24381632

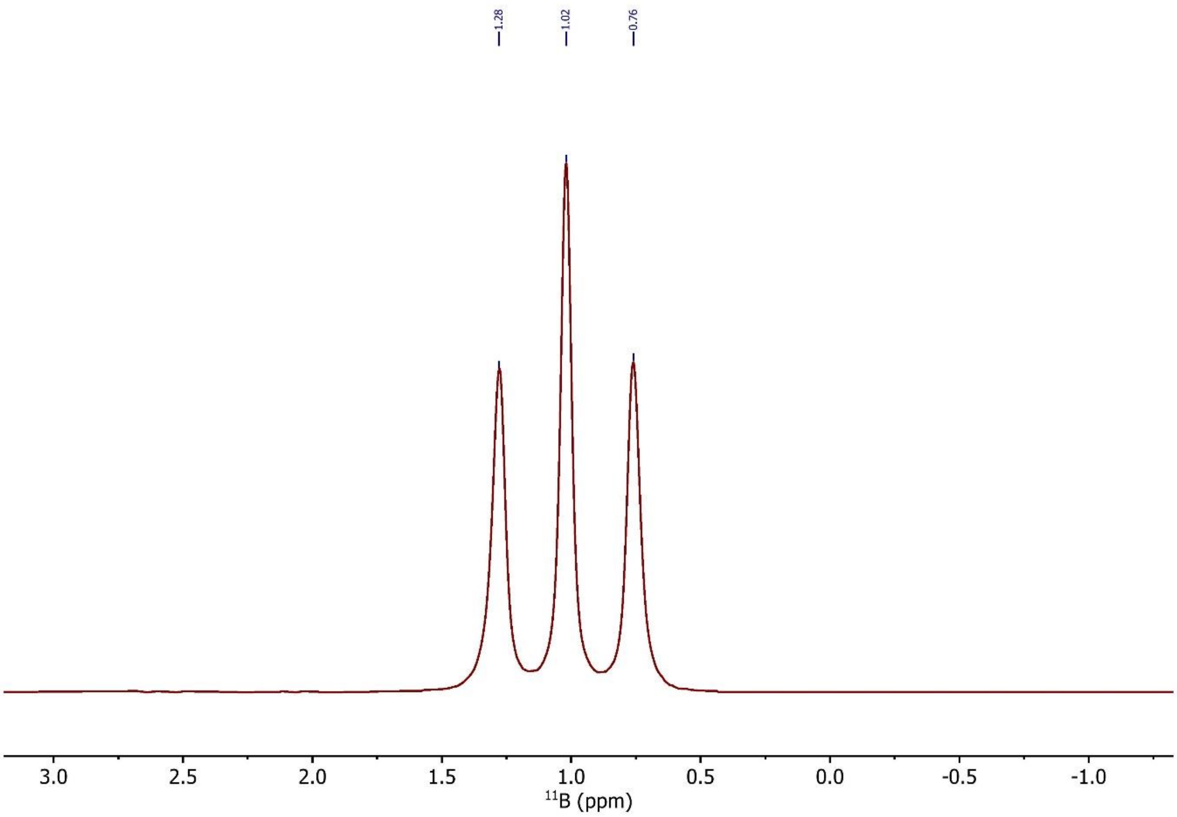

Figure S8. 160.5 MHz $^{11}B\{^{19}F\}$ NMR spectrum of compound **2** in PS/THF-d_8 after seven days of swelling. The splitting corresponds to ^{11}B RQC = –41.6 Hz.

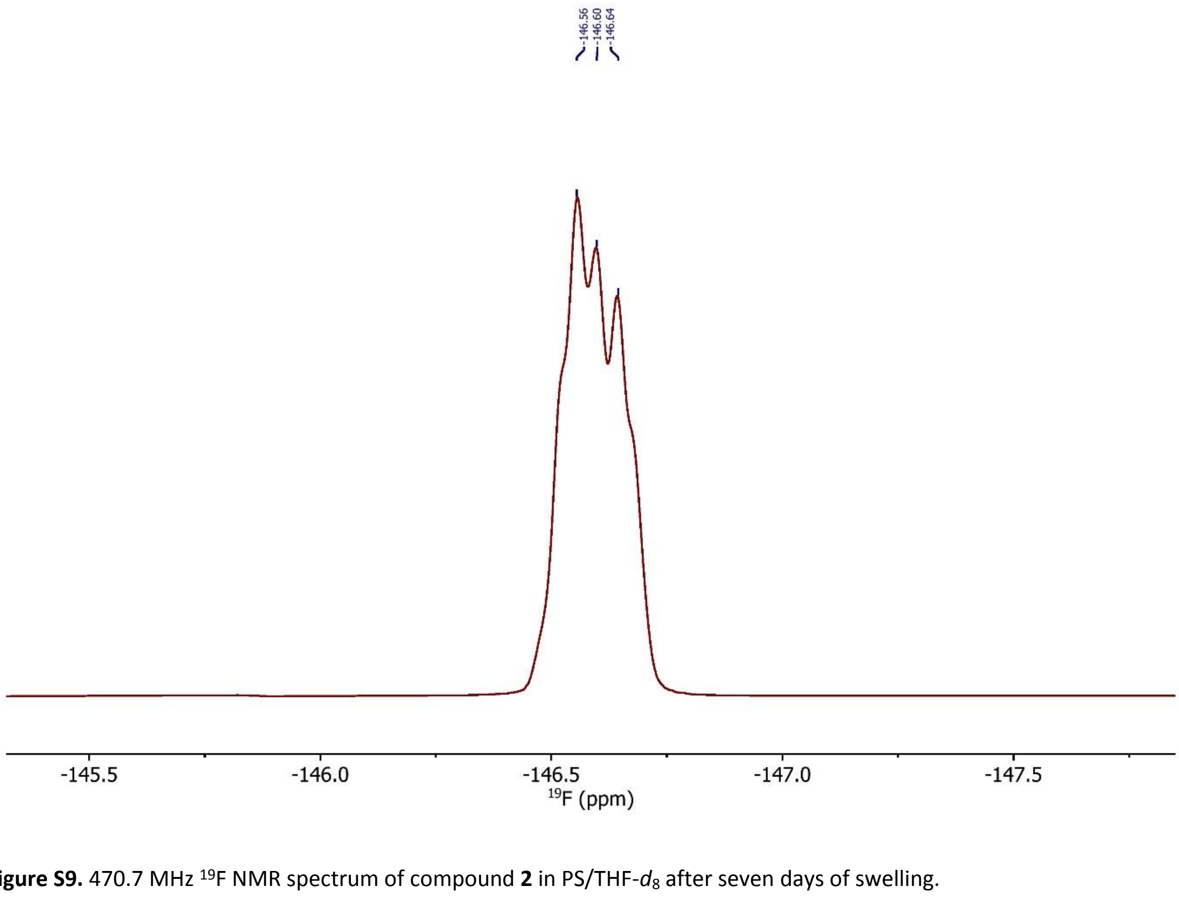

Figure S9. 470.7 MHz ^{19}F NMR spectrum of compound **2** in PS/THF-d_8 after seven days of swelling.

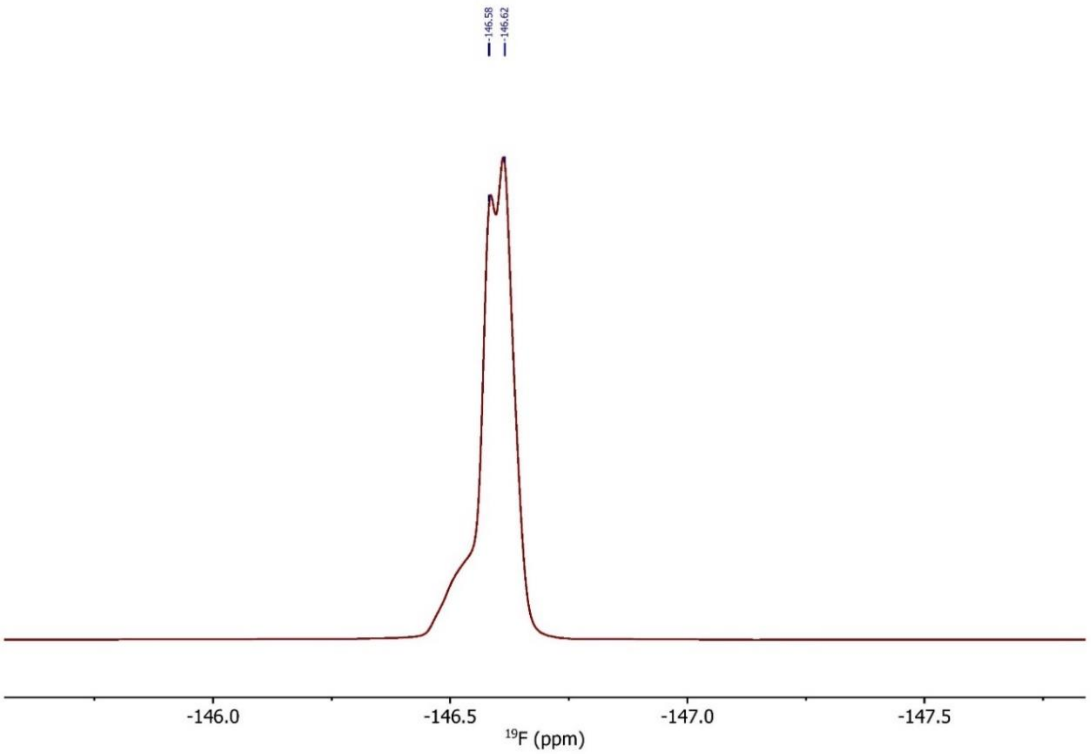

Figure S10. 470.7 MHz $^{19}F\{^{11}B\}$ NMR spectrum of compound **2** in PS/THF-d_8 after seven days of swelling. The splitting corresponds to $^{2}D_{F\text{-}F}$ = 12.1 Hz. The broad shoulder on the left side of the signal belongs to the ^{10}B isotopologue.

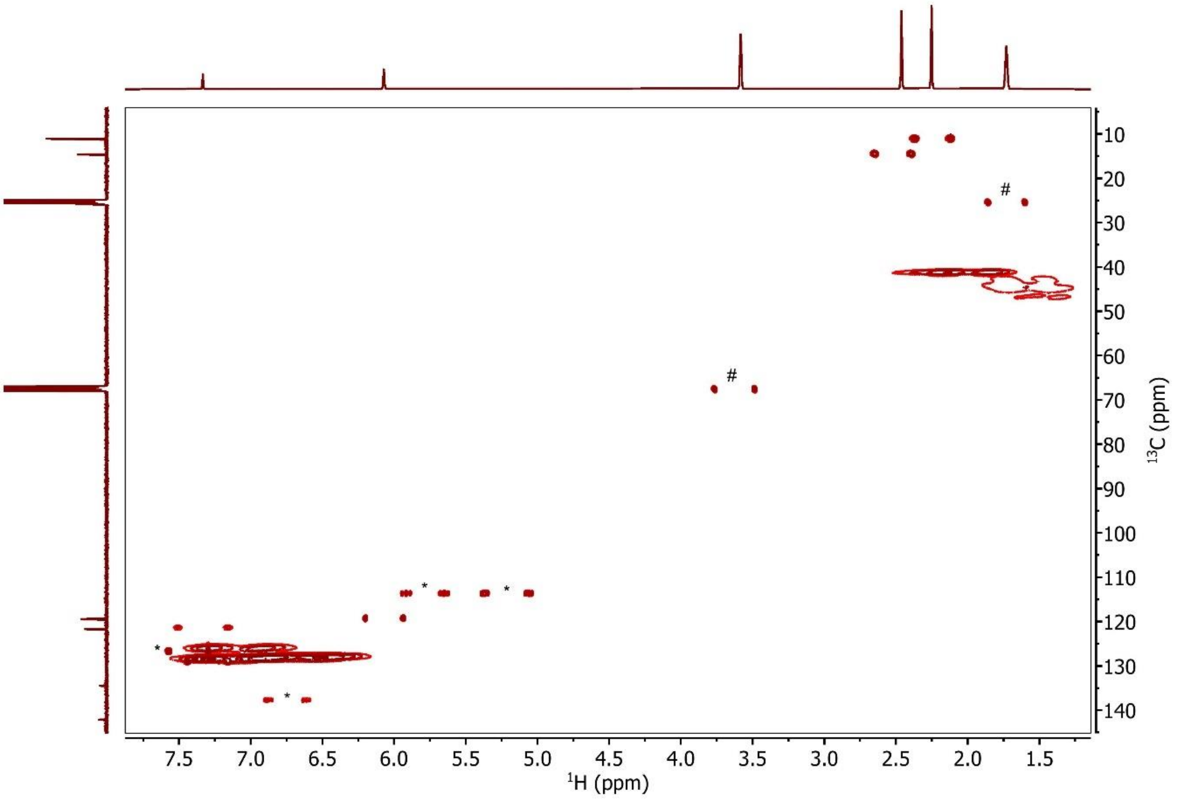

Figure S11. 500.3/125.8 MHz $^{1}H,^{13}C$ CLIP-HSQC spectrum of compound **2** in PS/THF-d_8 after seven days of swelling. Residual solvent signals are marked with #. Signals marked with * belong to unpolymerized styrene. The broad signals between $\delta(^{13}C)$ = 40-50 ppm and 125-130 ppm are from polystyrene. The 1D traces (^{1}H and ^{13}C) were taken from the isotropic spectra.

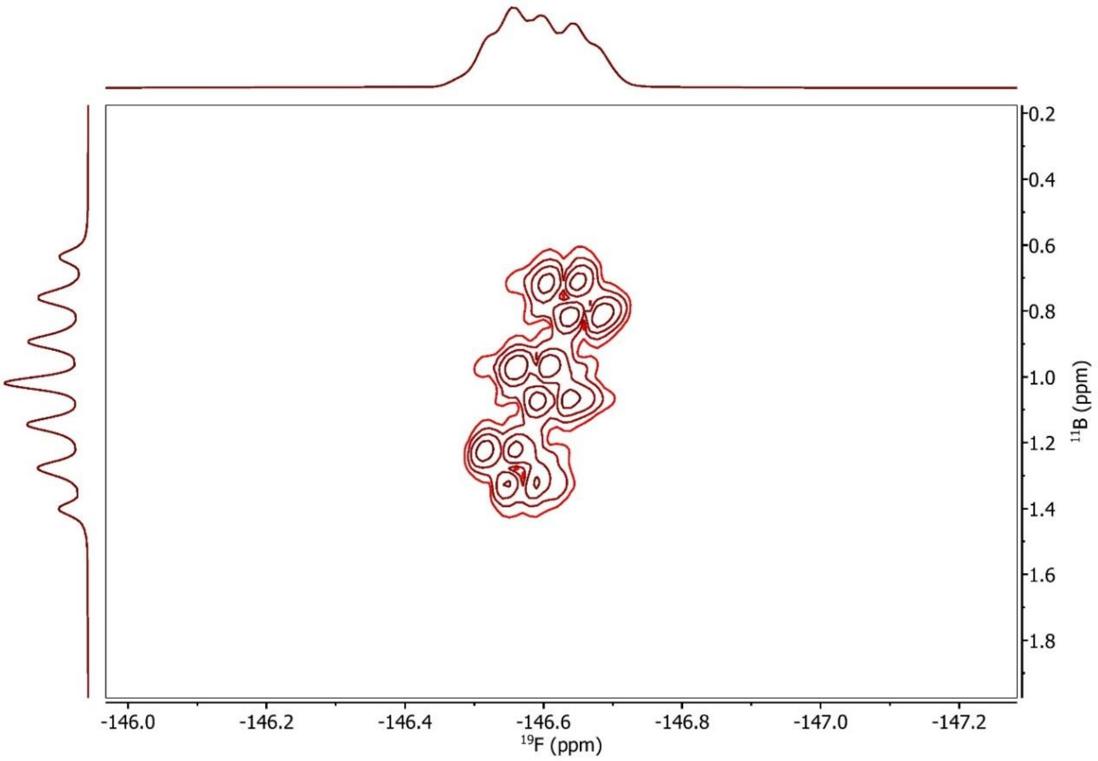

Figure S12. 470.7/160.5 MHz ^{19}F,^{11}B HMQC spectrum of compound **2** in PS/THF-d_8 after seven days of swelling with a ^{11}B flip angle of 30°. From the tilt of the signal, a negative ^{11}B RQC can be deduced.

NMR spectra of compound **3**

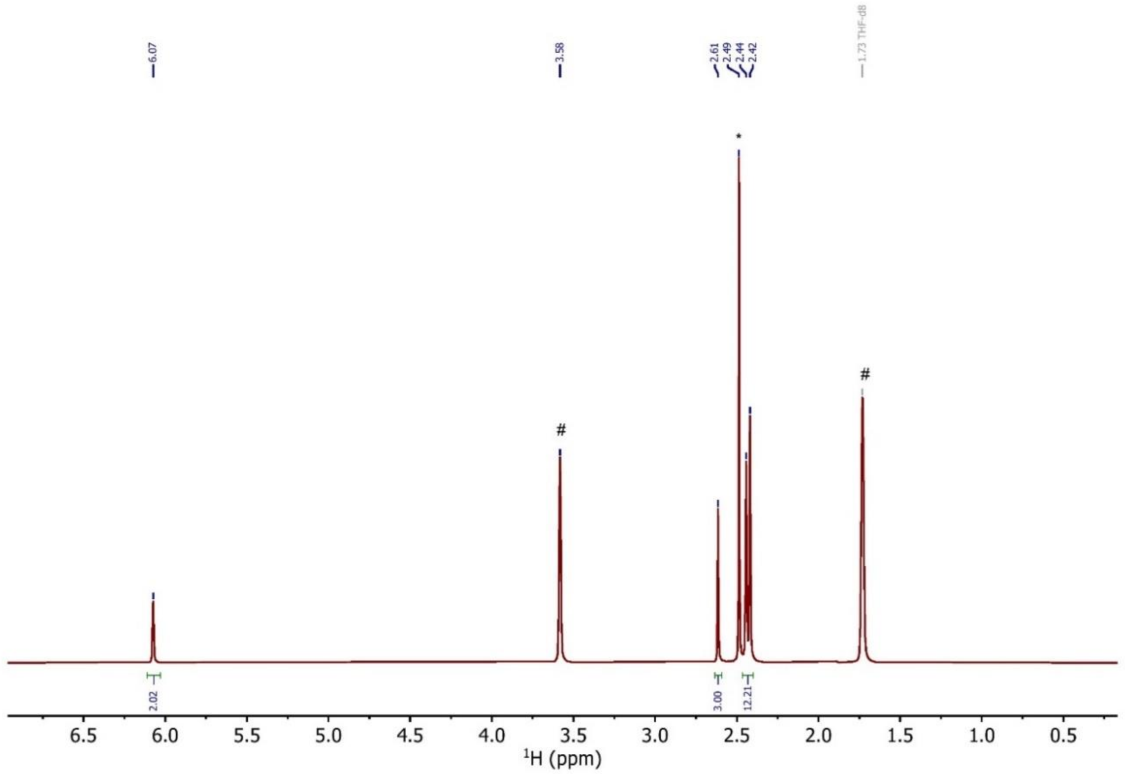

Figure S13. 400.3 MHz ^{1}H NMR spectrum of compound **3** in THF-d_8. Residual solvent = #, trace water = *.

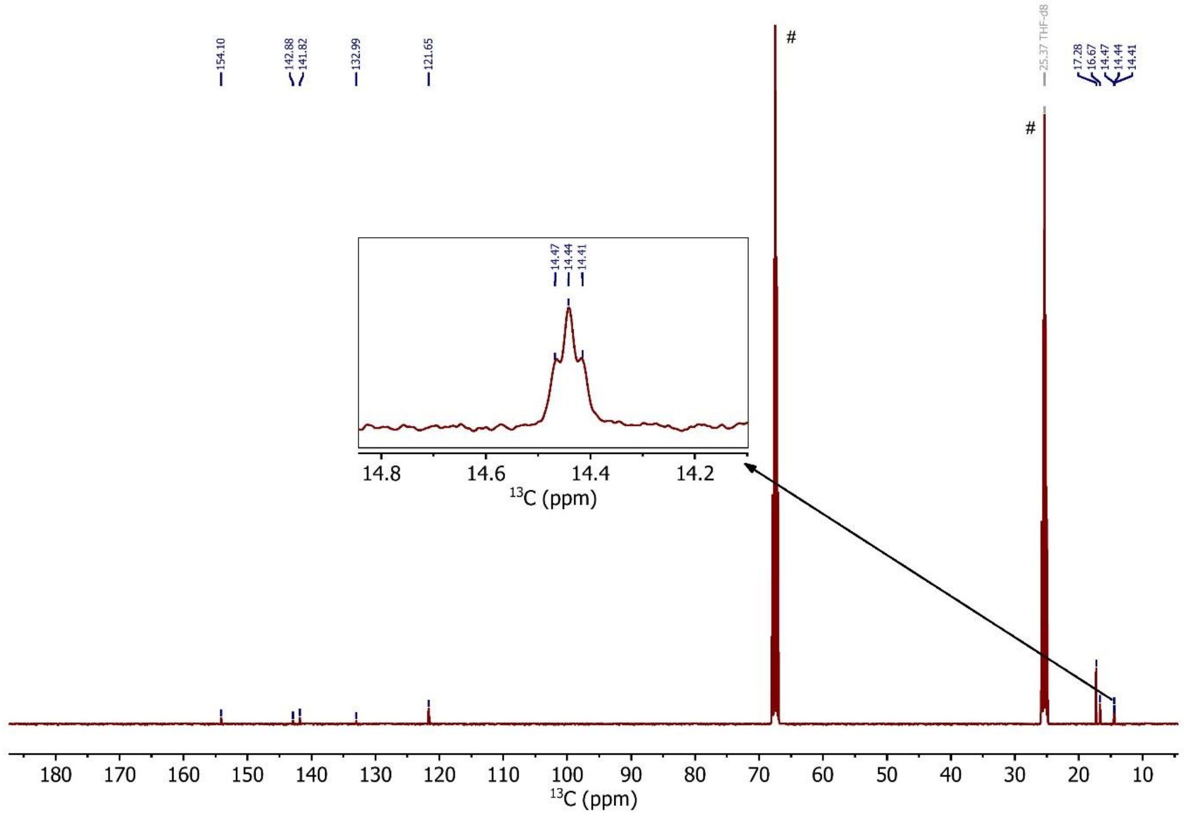

Figure S14. 100.7 $^{13}C\{^{1}H\}$ NMR spectrum of compound **3** in THF-d_8. Residual solvent signals are marked with #. The methyl groups close to the BF_2 group show a splitting of $J_{C\text{-}F}$ = 2.1 Hz.

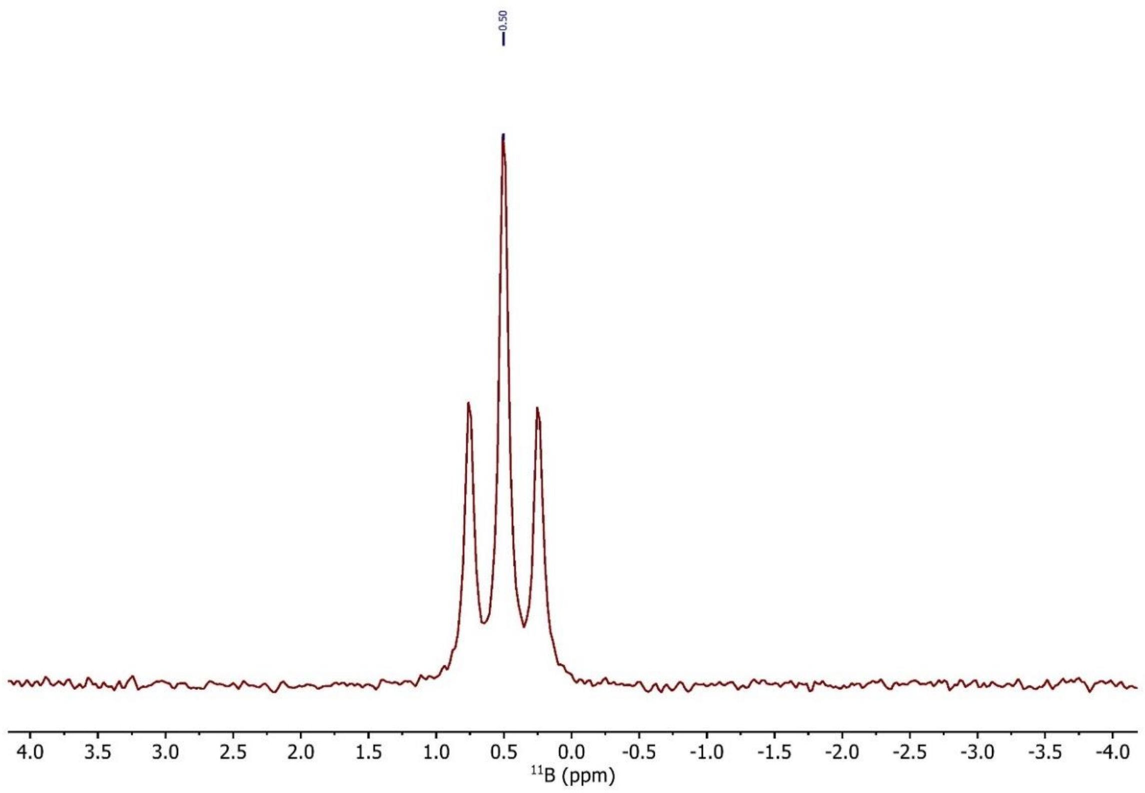

Figure S15. 128.4 MHz ^{11}B NMR spectrum of compound **3** in THF-d_8. The splitting corresponds to $^1J_{B\text{-}F}$ = –32.5 Hz.

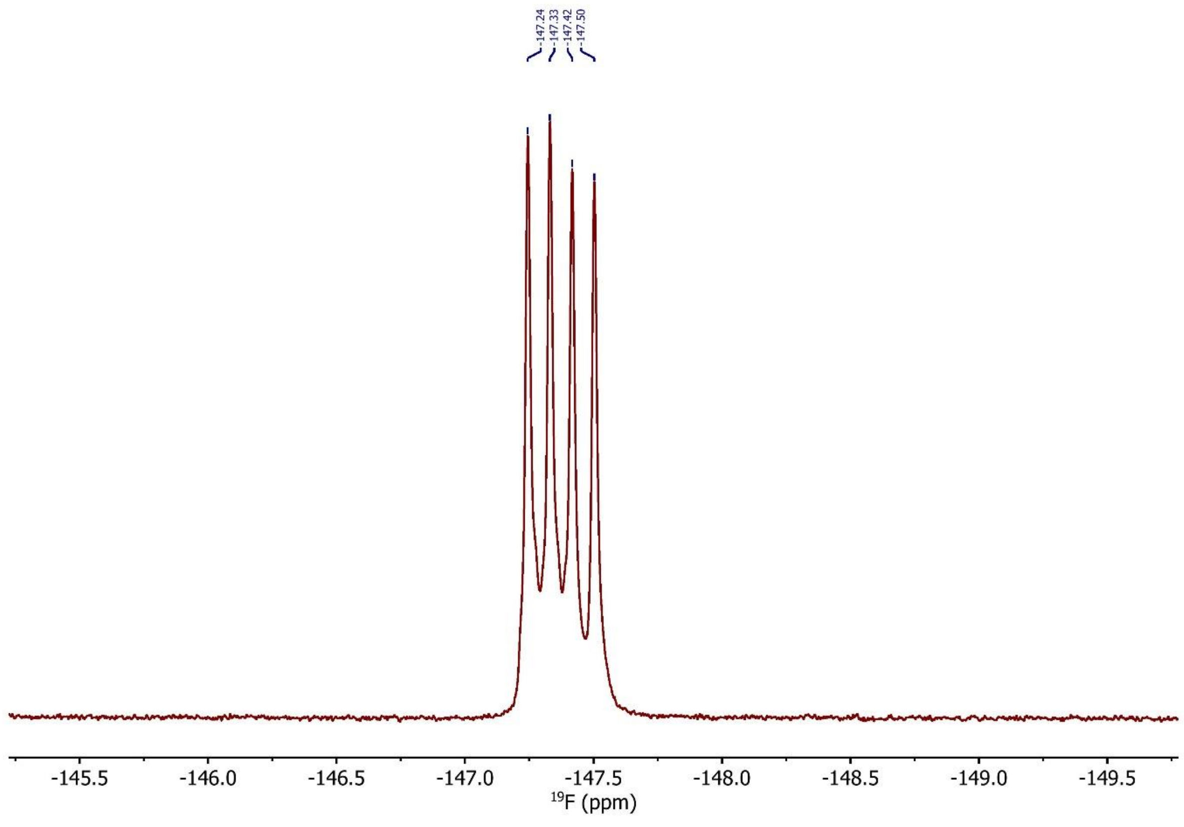

Figure S16. 376.6 MHz ^{19}F NMR spectrum of compound **3** in THF-d_8. The splitting corresponds to $^1J_{B\text{-}F}$ = –32.5 Hz.

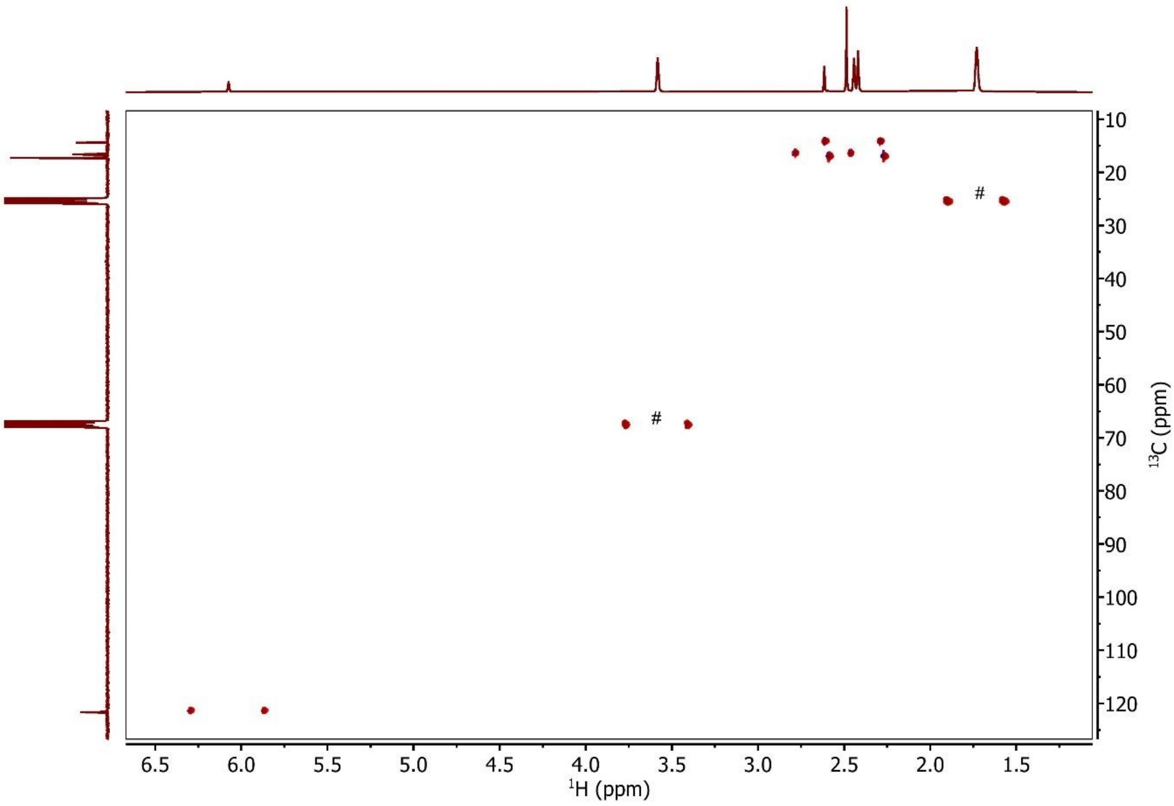

Figure S17. 400.3/100.7 MHz ^{1}H,^{13}C CLIP-HSQC spectrum of compound **3** in THF-d_8. Residual solvent signals are marked #.

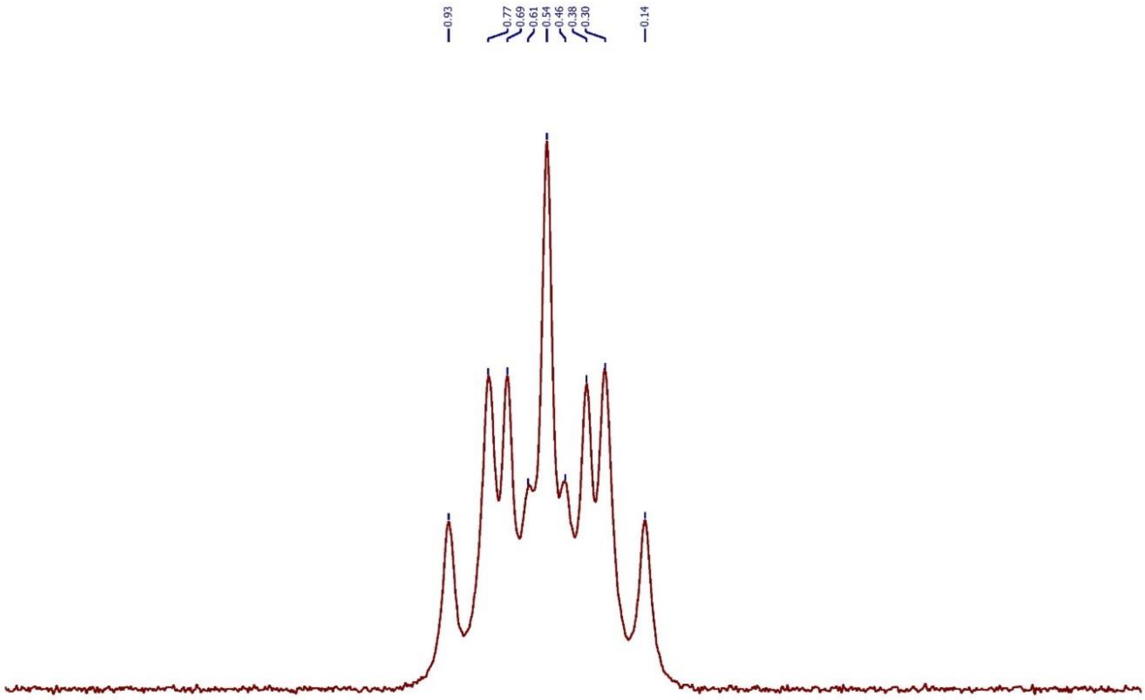

Figure S18. 160.5 MHz ^{11}B NMR spectrum of compound **3** in PS/THF-d_8 after seven days of swelling. The splittings correspond to $^{1}T_{B\text{-}F}$ = –25.5 Hz and ^{11}B RQC = –37.6 Hz.

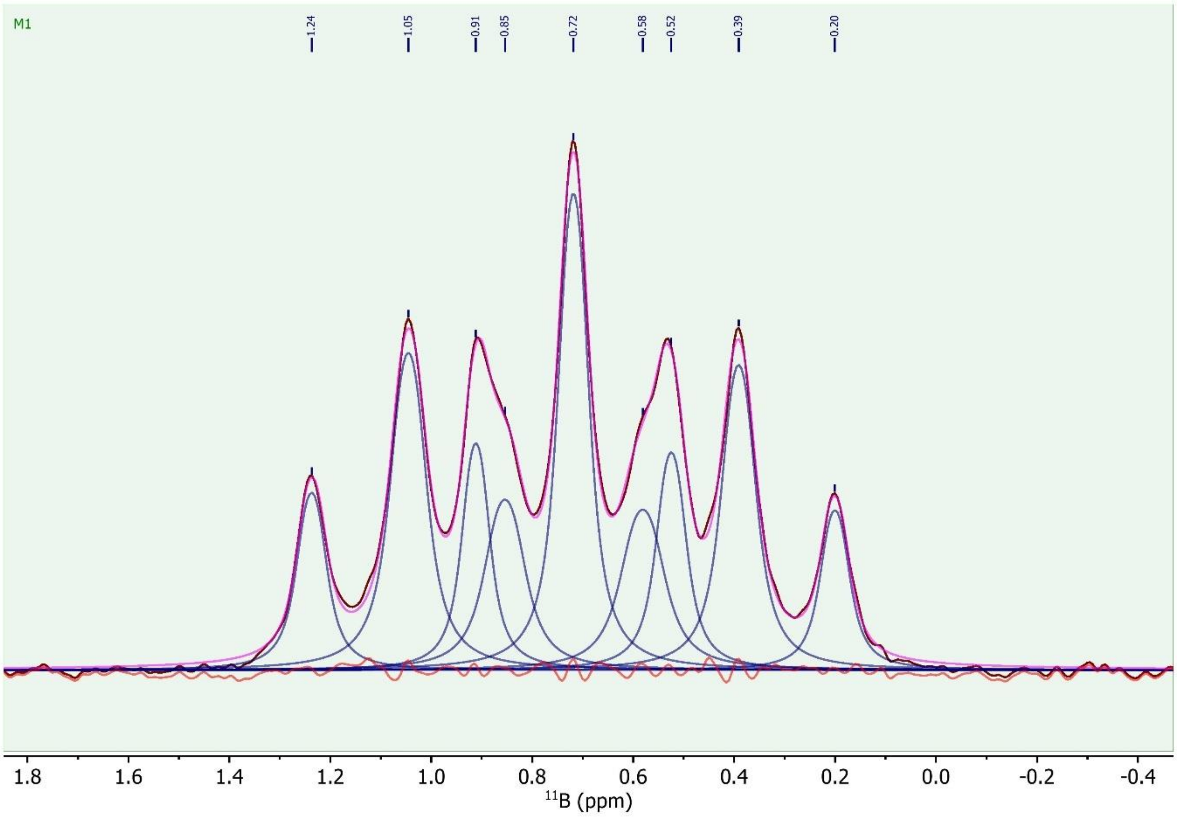

Figure S19. 128.4 MHz ^{11}B NMR spectrum of a 1.3 mm slice at the center of the NMR coil of compound **3** in PS/THF-d_8 after fifteen days of swelling with a lineshape fitting performed on the multiplet.

Table S2. Lineshape fitting parameters for the ^{11}B multiplet of compound **3** as shown in **Figure S19**. The L/G parameter was fixed to 0.75 for all signals. Widths include LB = 2 Hz from exponential multiplication.

δ (ppm)	Height	Width (Hz)	Area
1.237	405297	8.9	7939986
1.045	724775	10.8	17157240
0.912	518544	8.3	9448034
0.854	389525	12.0	10290337
0.718	1089146	9.1	21719691
0.581	366625	12.9	10422492
0.525	497388	9.0	9801340
0.391	697388	10.2	15699893
0.200	365247	8.7	6994905

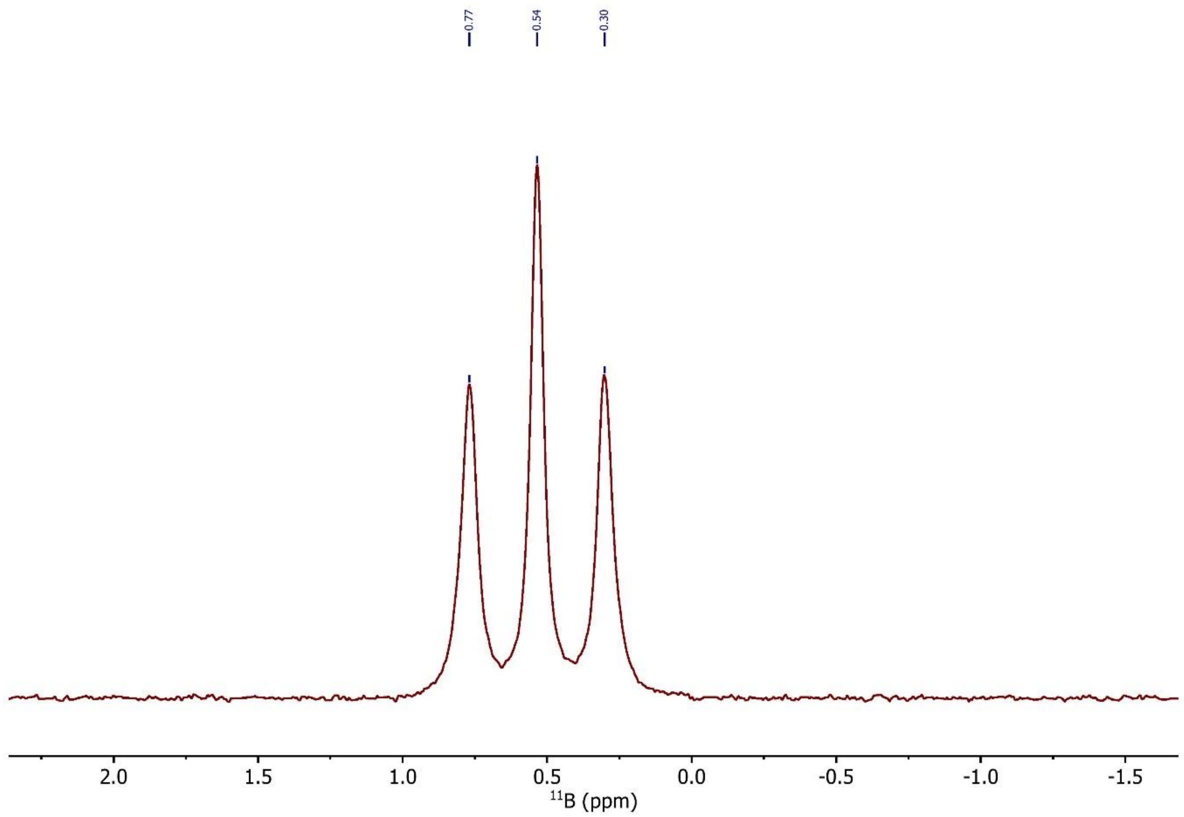

Figure S20. 160.5 MHz $^{11}B\{^{19}F\}$ NMR spectrum of compound **3** in PS/THF-d_8 after seven days of swelling. The splitting corresponds to ^{11}B RQC = –37.6 Hz.

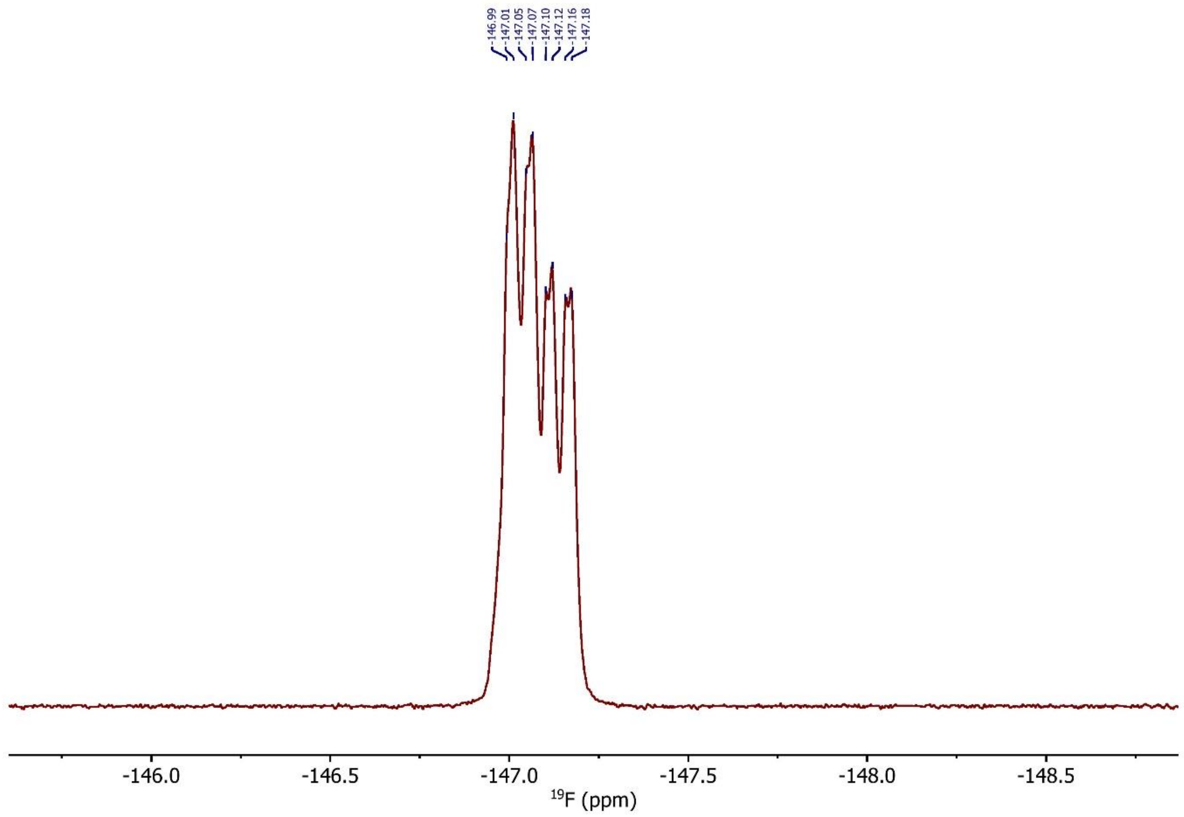

Figure S21. 470.7 MHz ^{19}F NMR spectrum of compound **3** in PS/THF-d_8 after seven days of swelling.

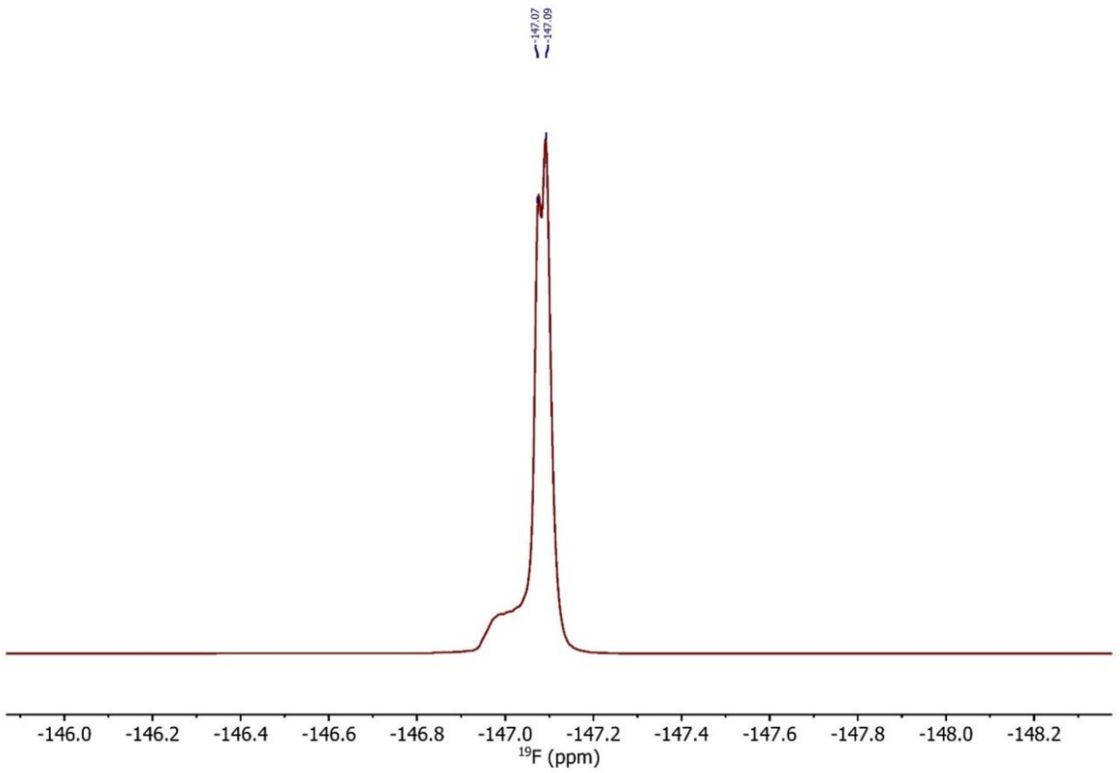

Figure S22. 470.7 MHz $^{19}F\{^{11}B\}$ NMR spectrum of compound **3** in PS/THF-d_8 after seven days of swelling. The splitting corresponds to $^{2}D_{F\text{-}F}$ = 7.1 Hz. The broad shoulder on the left side of the signal belongs to the ^{10}B isotopologue.

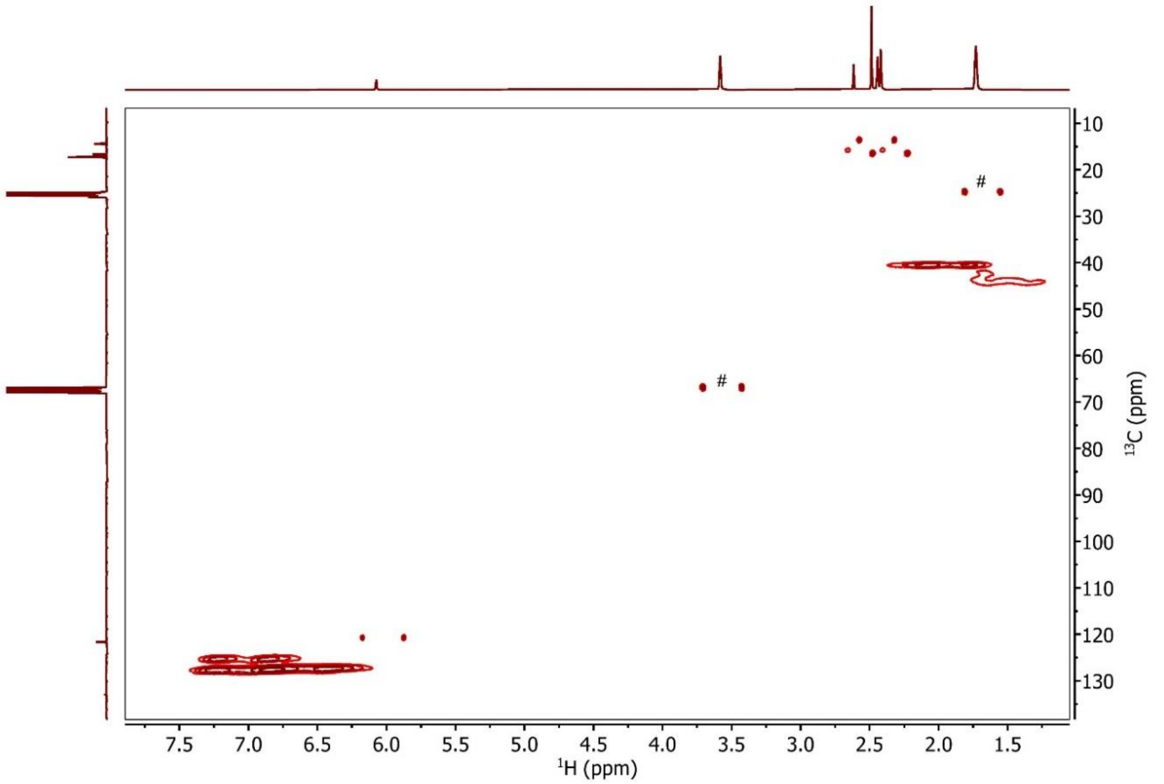

Figure S23. 500.3/125.8 MHz $^{1}H,^{13}C$ CLIP-HSQC spectrum of compound **3** in PS/THF-d_8 after seven days of swelling. Residual solvent signals are marked with #. The broad signals between $\delta(^{13}C)$ = 40-50 ppm and 125-130 ppm are of polystyrene. The 1D traces (^{1}H and ^{13}C) were taken from the isotropic spectra.

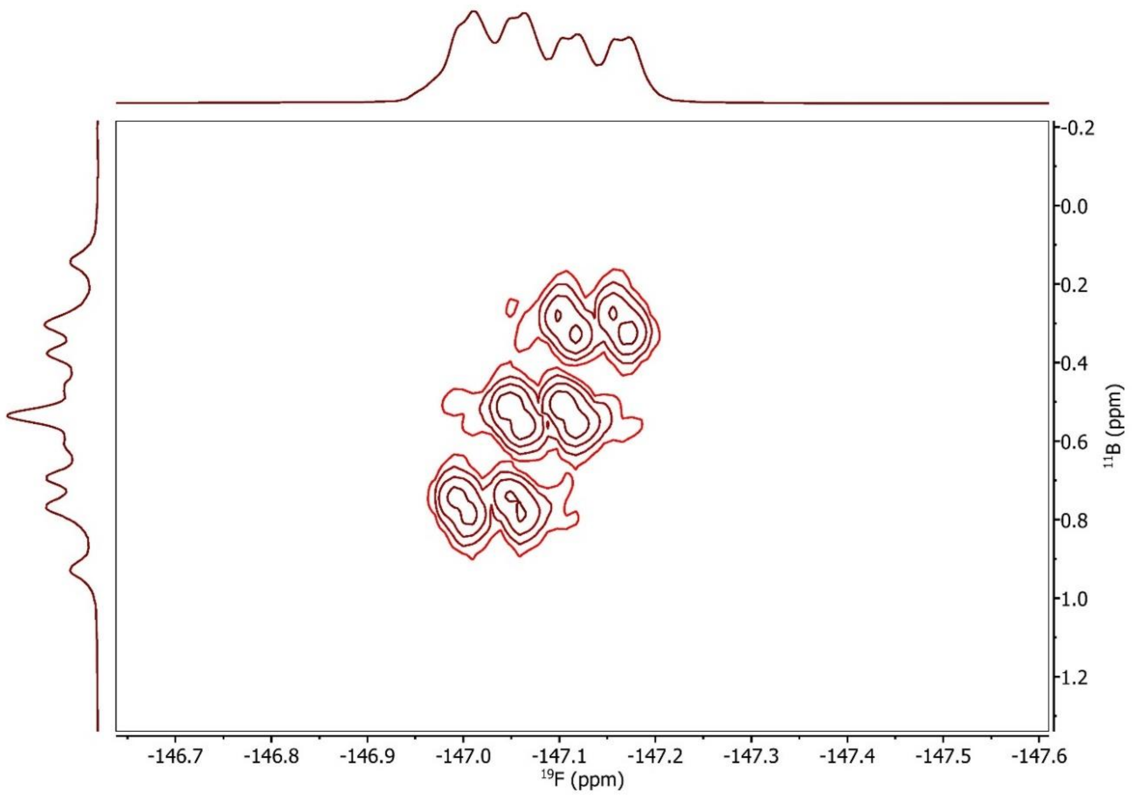

Figure S24. 470.7/160.5 MHz ^{19}F,^{11}B HMQC spectrum of compound **3** in PS/THF-d_8 after seven days of swelling with a ^{11}B flip angle of 30°. From the tilt of the signal, a negative ^{11}B RQC can be deduced.

NMR spectra of compound **6**

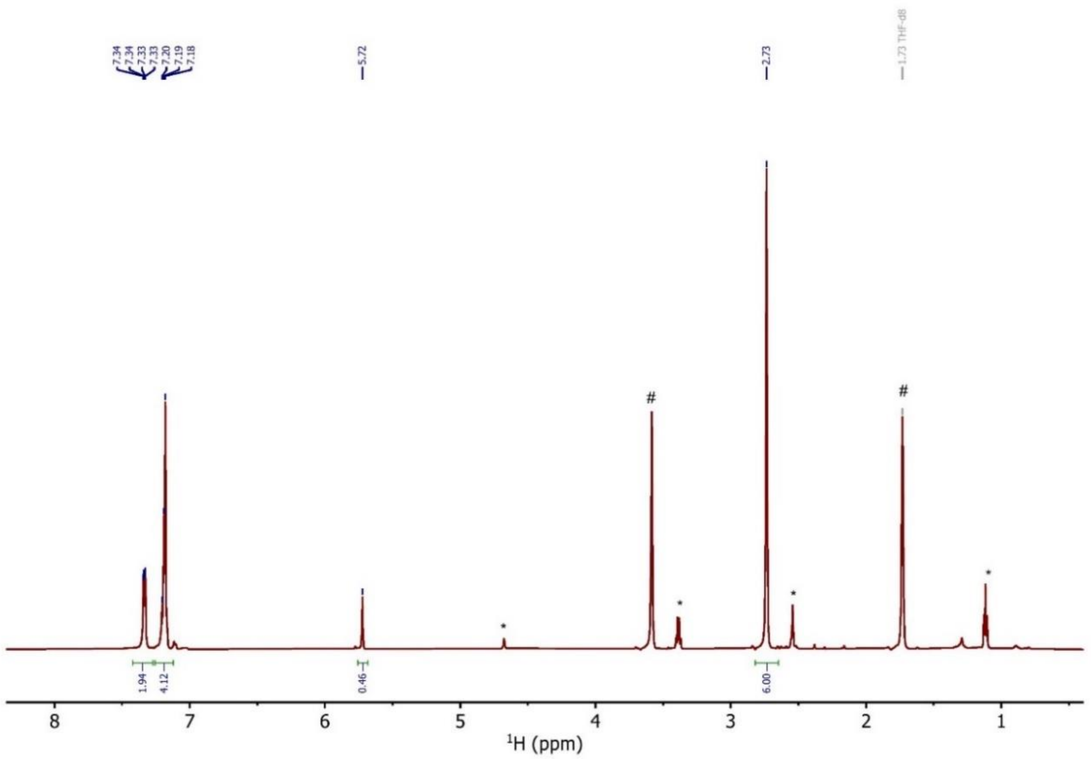

Figure S25. 600.3 MHz ^{1}H NMR spectrum of compound **6** in THF-d_8. The residual solvent signals are marked with #. The signals marked with * stem from residual ligand and diethyl ether.

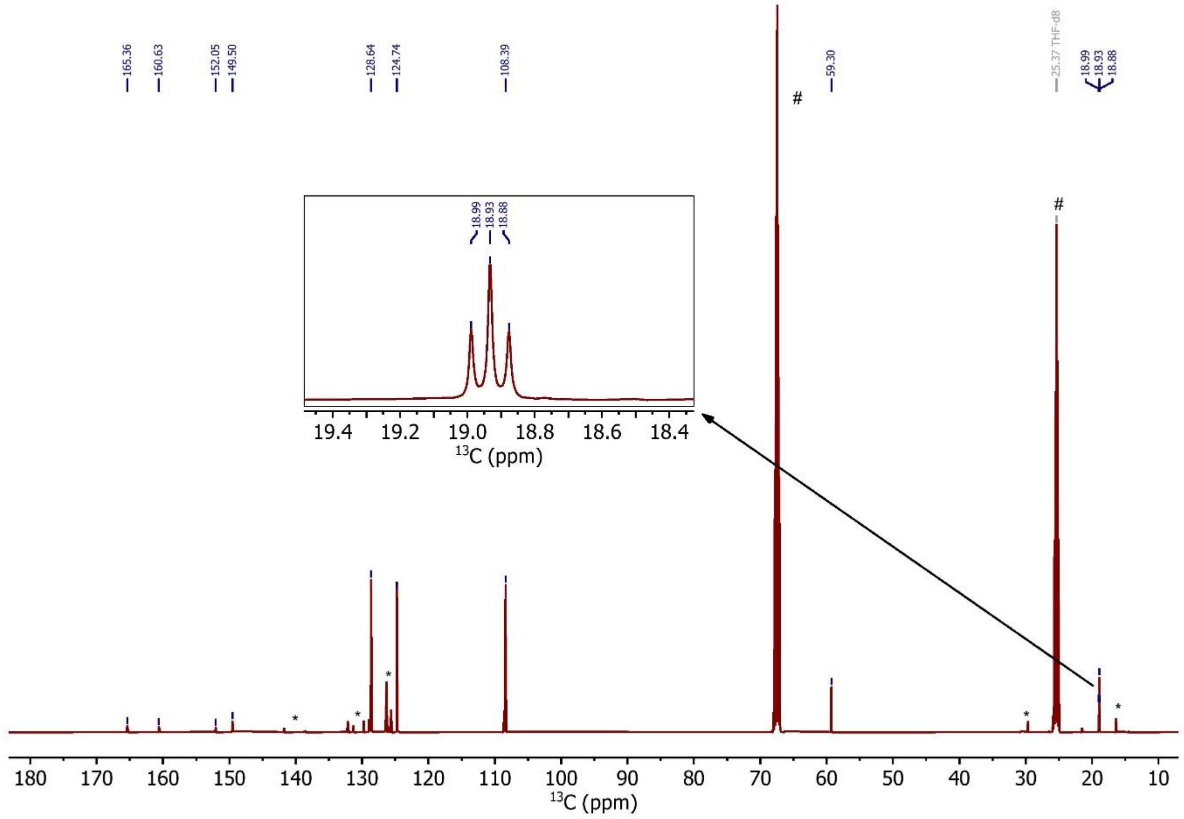

Figure S26. 125.8 MHz $^{13}C\{^{1}H\}$ NMR spectrum of compound **6** in THF-d_8. Residual solvent signals are marked with #. The signals marked with * stem from residual ligand. The methyl groups show a splitting of $J_{C\text{-}F}$ = 7.0 Hz.

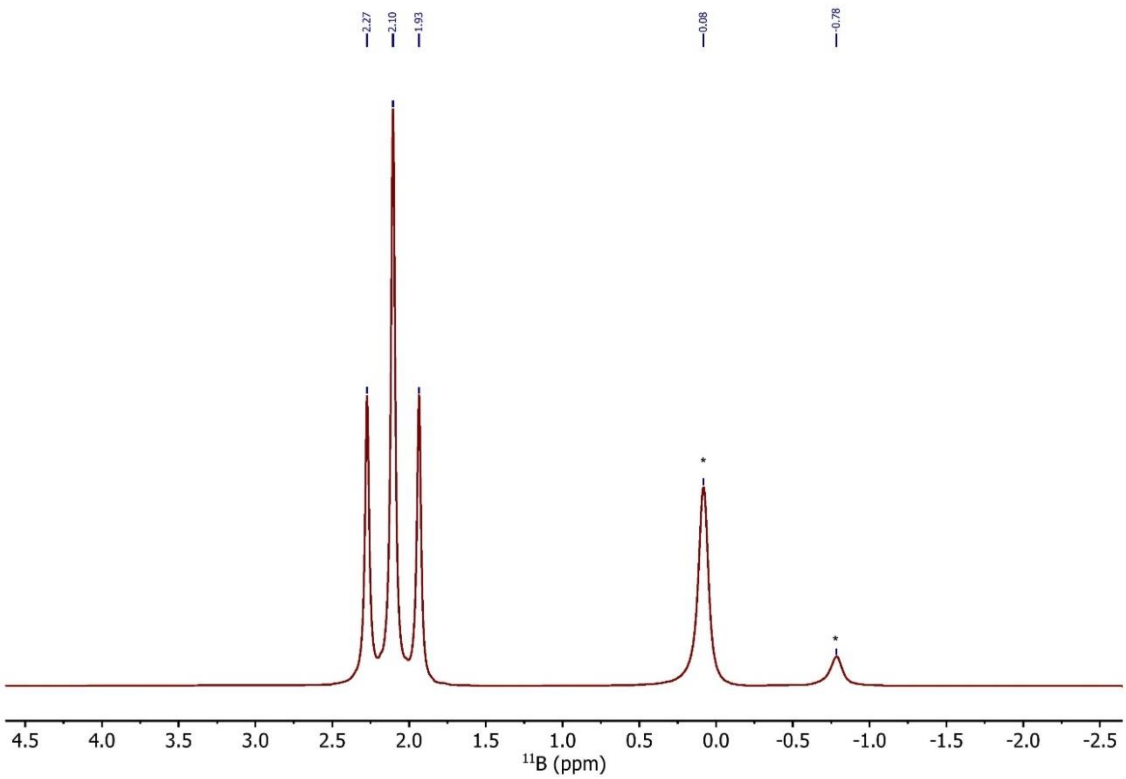

Figure S27. 160.5 MHz ^{11}B NMR spectrum of compound **6** in THF-d_8. The signals marked with * belong to impurities (BF_4^- and $BF_3 \cdot OEt_2$). The splitting corresponds to ($^1J_{B\text{-}F}$ = –27.1 Hz).

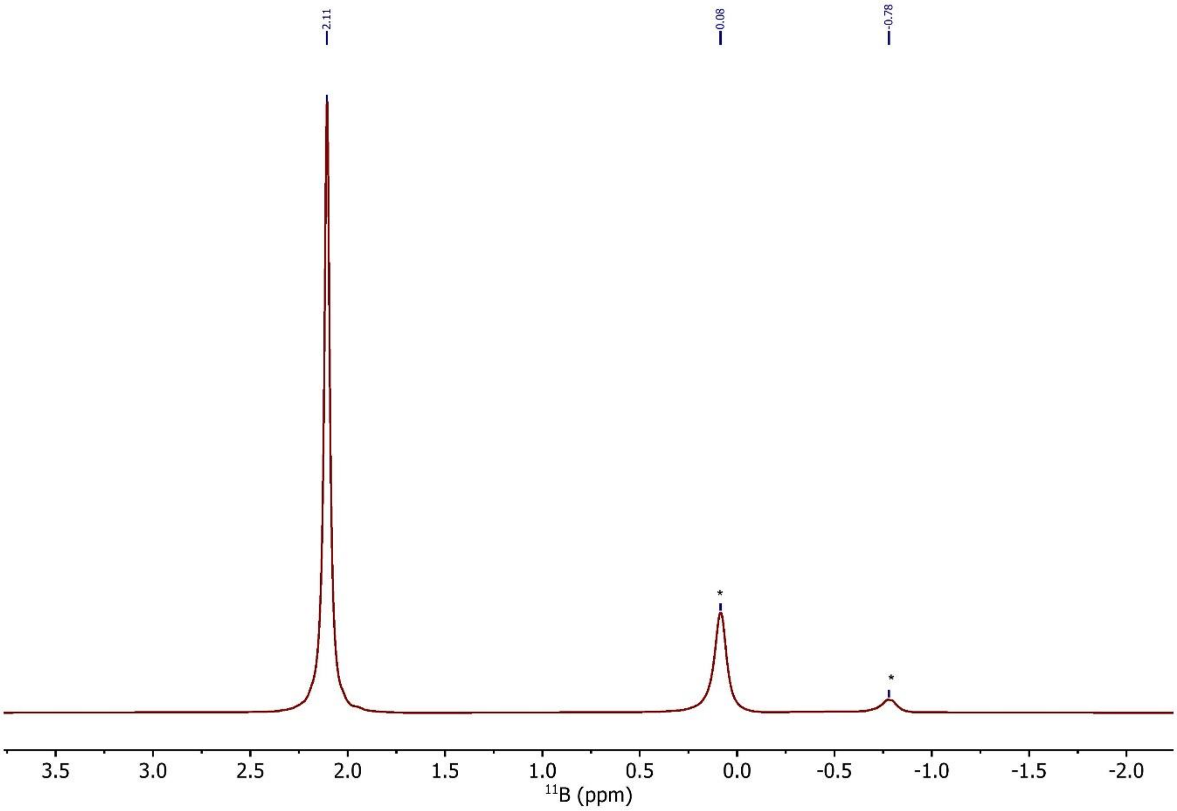

Figure S28. 160.5 MHz $^{11}B\{^{19}F\}$ NMR spectrum of compound **6** in THF-d_8. The signals marked with * belong to impurities (BF_4^- and $BF_3 \cdot OEt_2$).

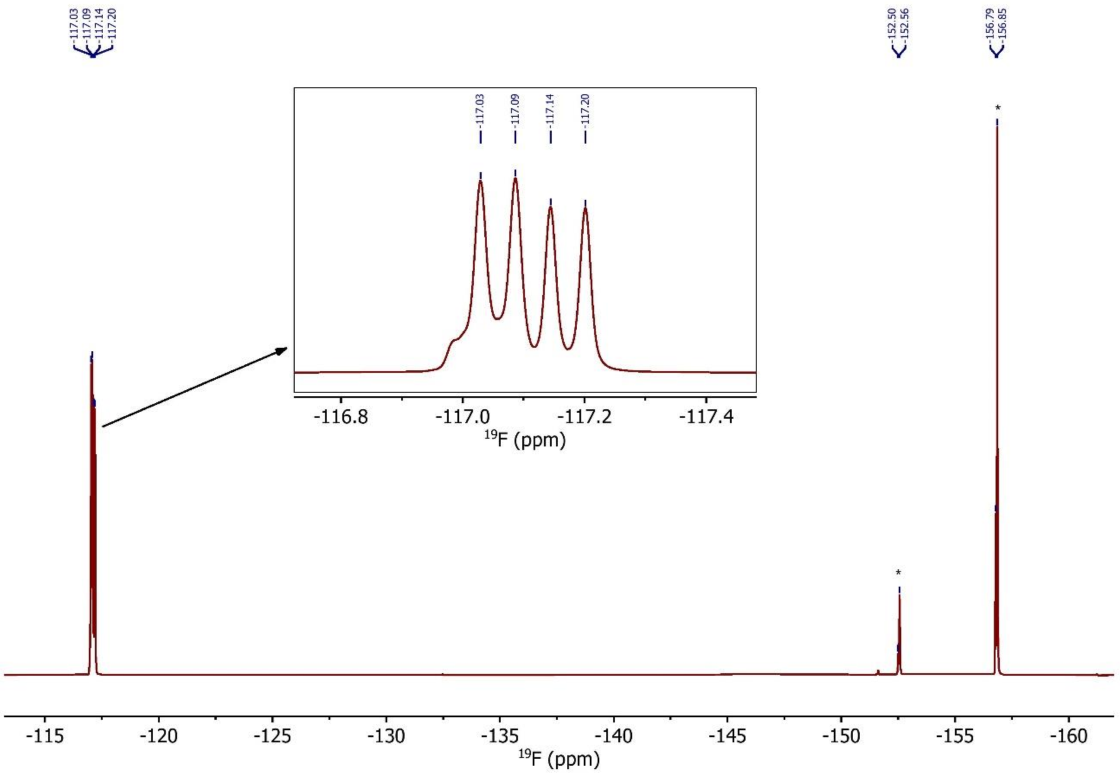

Figure S29. 470.7 MHz ^{19}F NMR spectrum of compound **6** in THF-d_8. The signals marked with * belong to impurities ($BF_3{\cdot}OEt_2$ and BF_4^-). The splitting corresponds to $^{1}J_{B\text{-}F} = -27.1$ Hz.

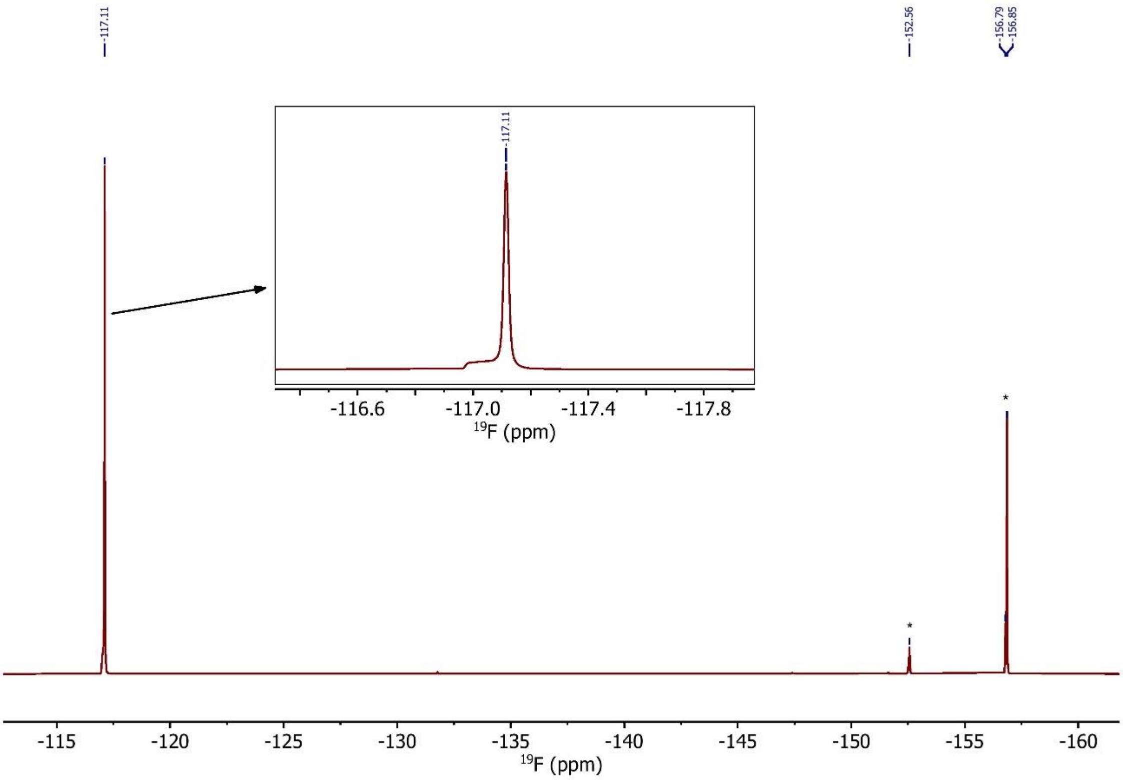

Figure S30. 564.7 MHz $^{19}F\{^{11}B\}$ NMR spectrum of compound **6** in THF-d_8. The signals marked with * belong to impurities ($BF_3{\cdot}OEt_2$ and BF_4^-). The broad shoulder on the left side of the signal belongs to the ^{10}B isotopologue.

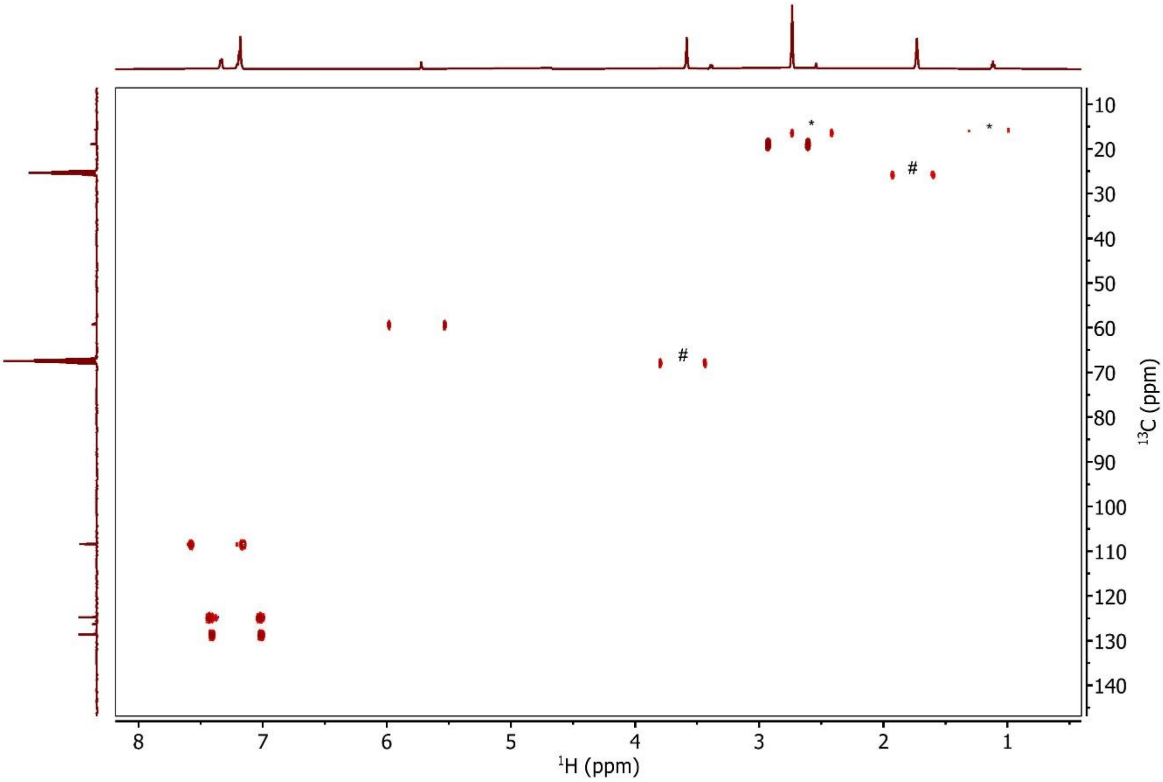

Figure S31. 400.3/100.7 MHz ^{1}H,^{13}C CLIP-HSQC spectrum of compound **6** in THF-d_8. Residual solvent signals are marked with #. The signals marked with * are from impurities.

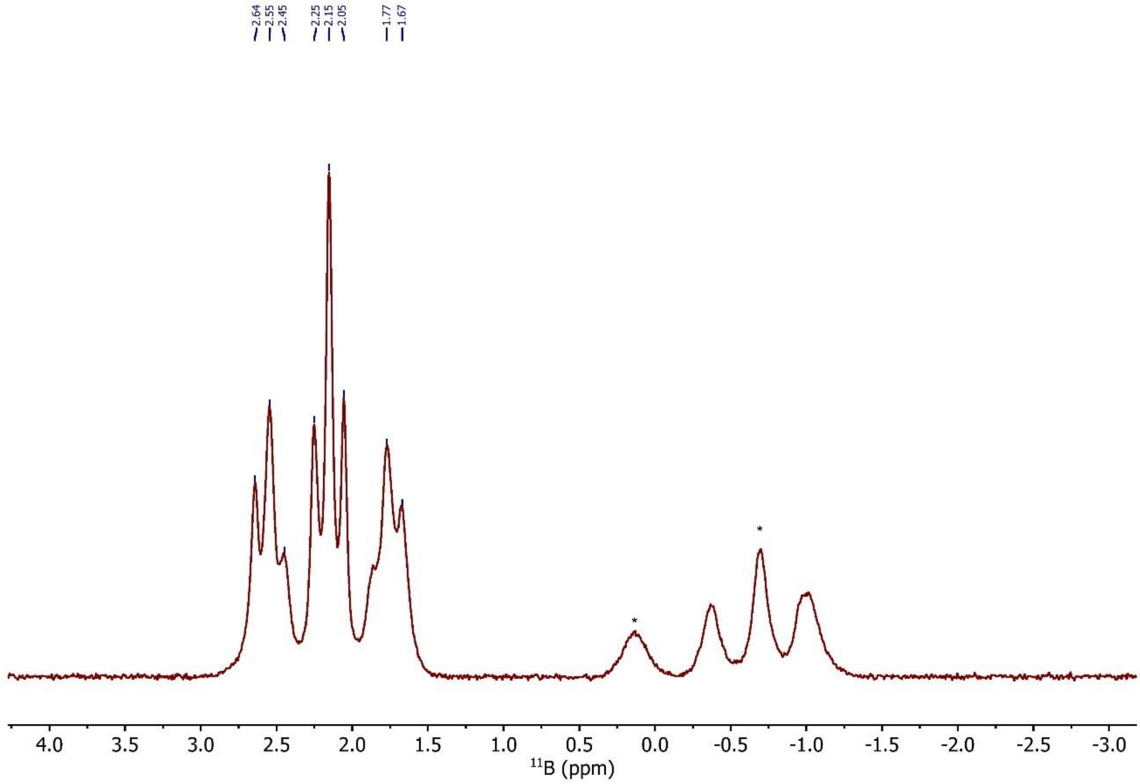

Figure S32. 160.5 MHz ^{11}B NMR spectrum of compound **6** in PS/THF-d_8 after seven days of swelling. The signals marked with * are from impurities (BF_4^- and $BF_3 \cdot OEt_2$). The splittings correspond to $^{1}T_{B\text{-}F} = -15.8$ Hz and ^{11}B RQC = 63.0 Hz.

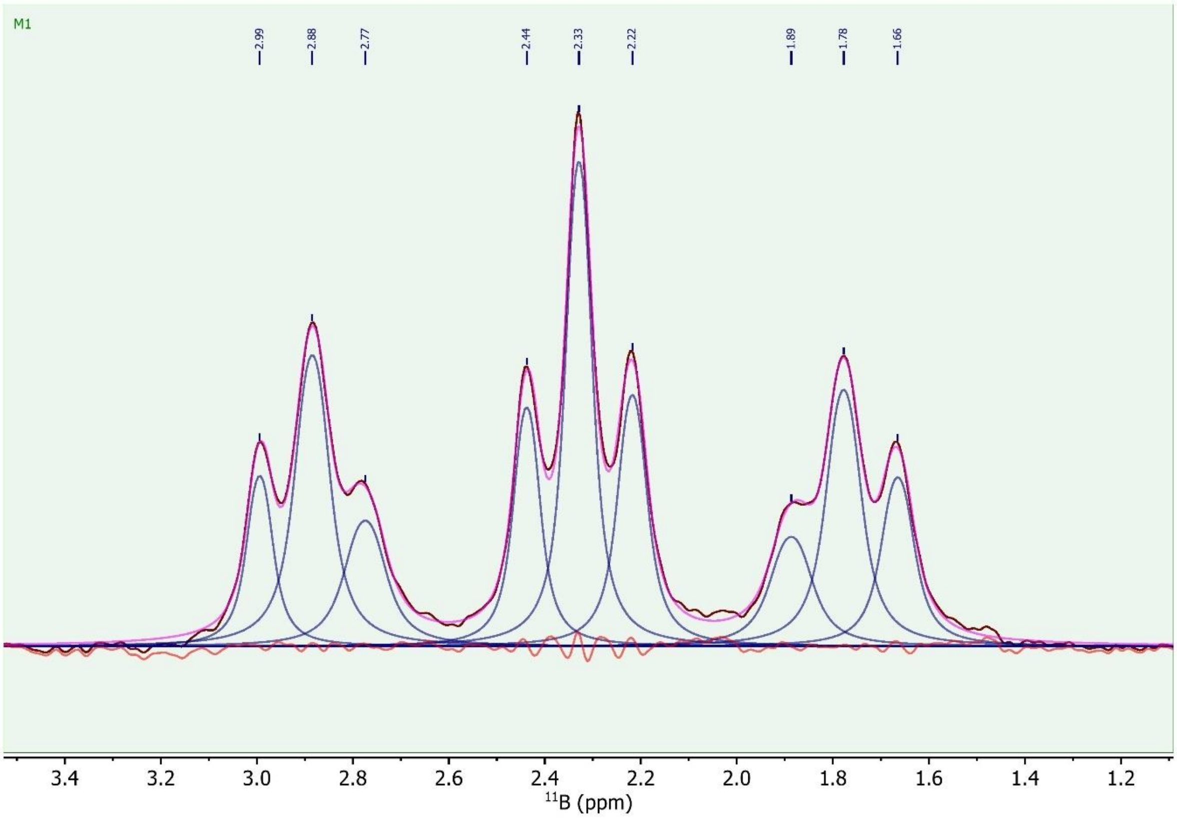

Figure S33. 128.4 MHz ^{11}B NMR spectrum of a 1.3 mm slice at the center of the NMR coil of compound **6** in PS/THF-d_8 after fifteen days of swelling with a lineshape fitting performed on the multiplet.

Table S3. Lineshape fitting parameters for the ^{11}B multiplet of compound **6** as shown in **Figure S33**. The L/G parameter was fixed to 0.75 for all signals. Widths include LB = 2 Hz from exponential multiplication.

δ (ppm)	Height	Width (Hz)	Area
2.994	503130	8.4	9256359
2.884	858839	11.0	20694924
2.774	371249	13.1	10665861
2.438	705398	8.4	13104190
2.329	1430921	8.4	26313950
2.218	741783	9.4	15280460
1.887	323516	13.2	9416378
1.778	756922	11.1	18427680
1.665	498958	10.4	11417261

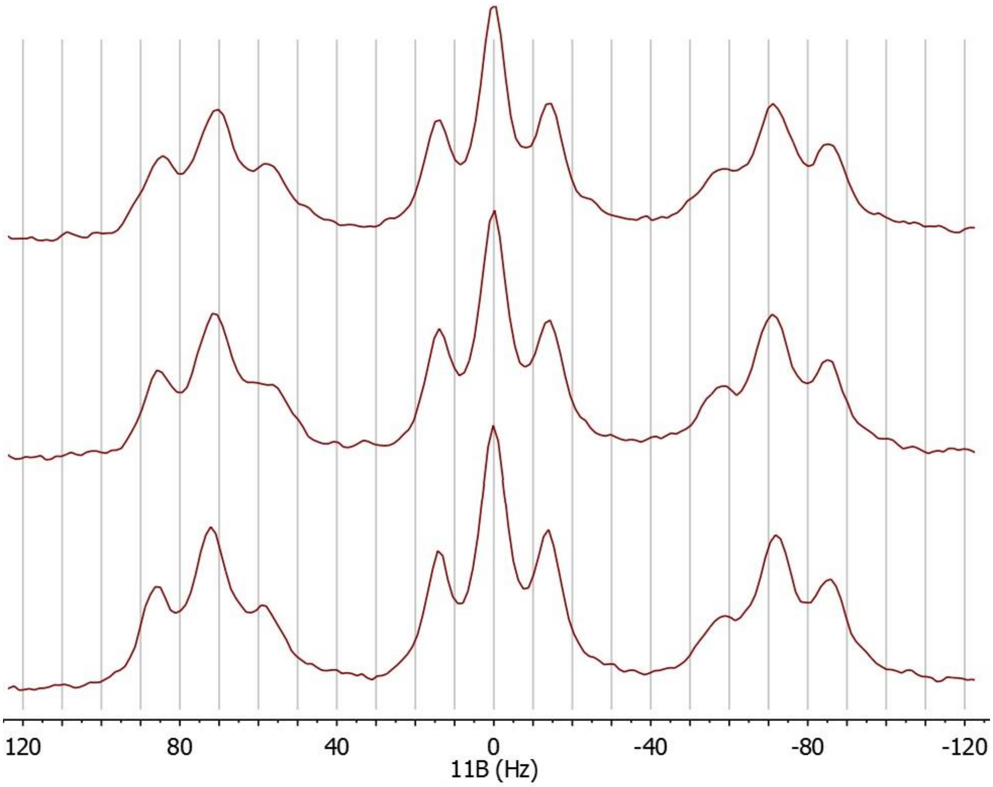

Figure S34. 128.4 MHz 1.3 mm slice-selective ^{11}B NMR spectra of compound **6** in PS/THF-d_8 after fifteen days of swelling. The spectra were recorded at positions +0.5 mm (top), 0 mm (center) and –0.5 mm relative to the center of the coil.

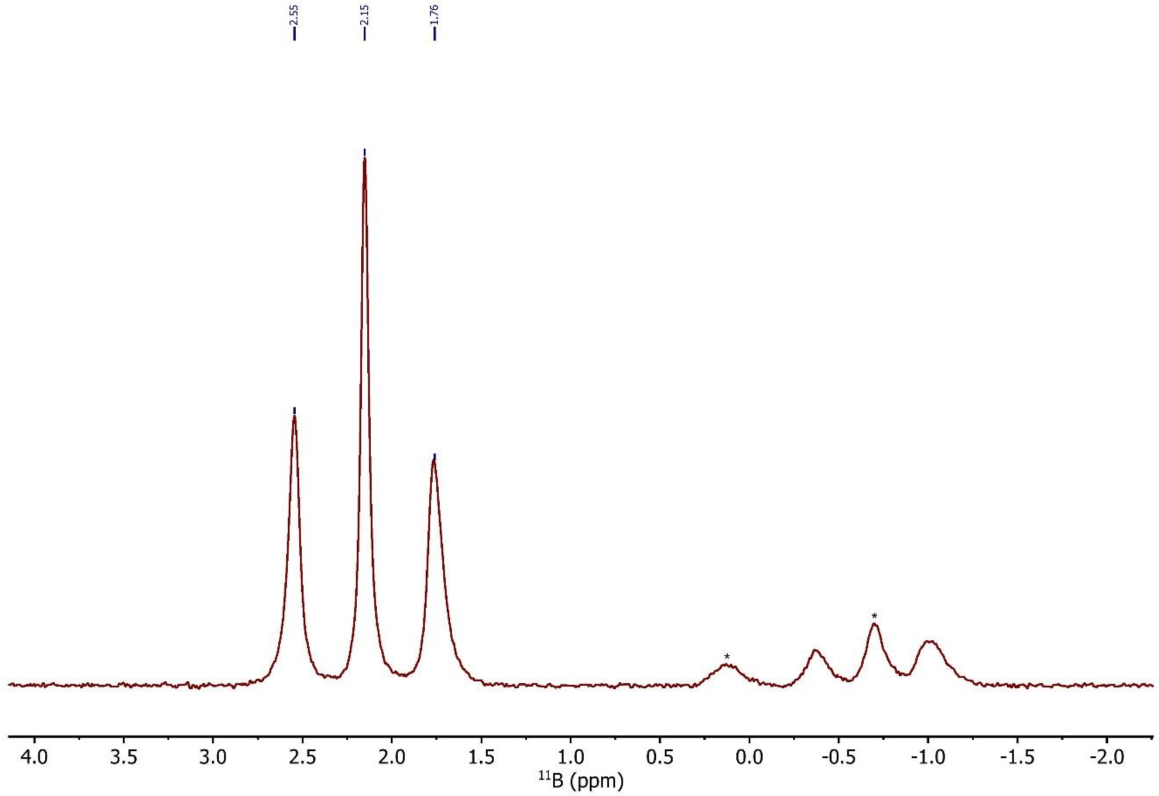

Figure S35. 160.5 MHz $^{11}B\{^{19}F\}$ NMR spectrum of compound **6** in PS/THF-d_8 after seven days of swelling. The signals marked with * are impurities (BF_4^- and $BF_3{\cdot}OEt_2$). The splitting corresponds to ^{11}B RQC = 63.0 Hz.

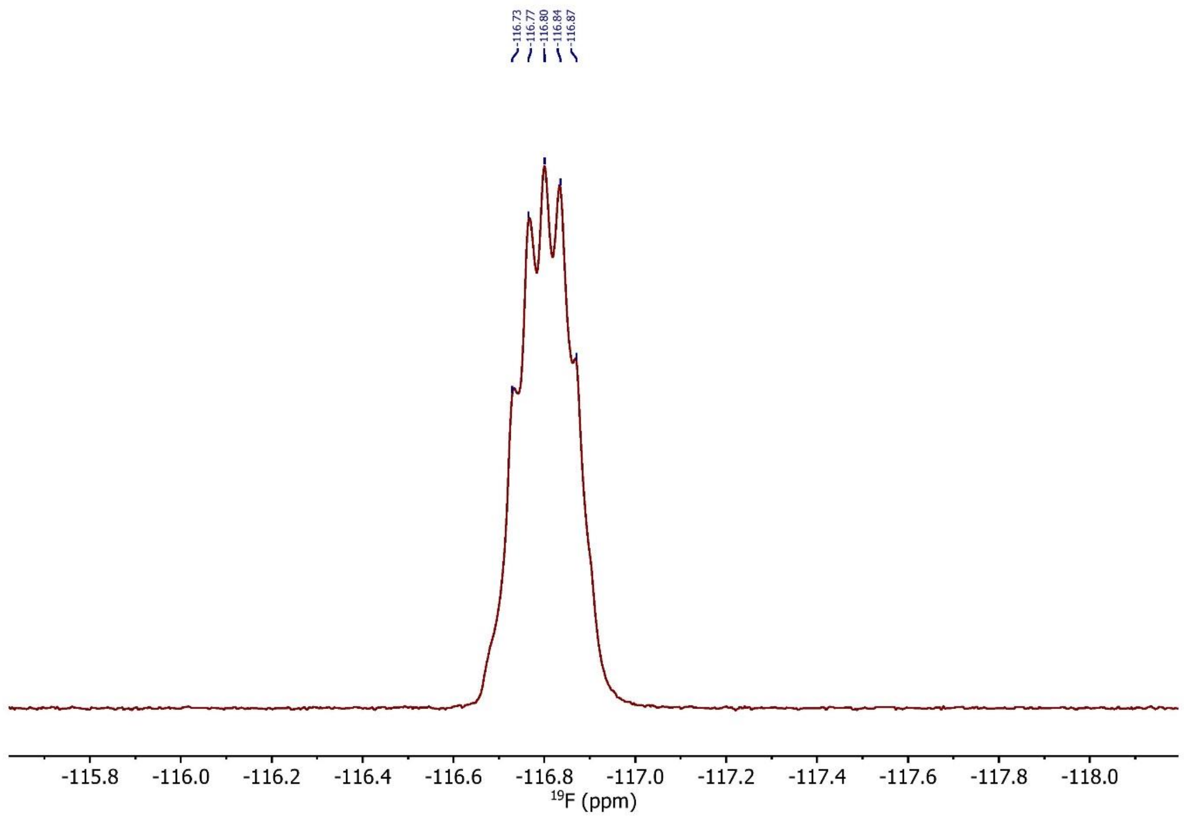

Figure S36. 470.7 MHz ^{19}F NMR spectrum of compound **6** in PS/THF-d_8 after seven days of swelling.

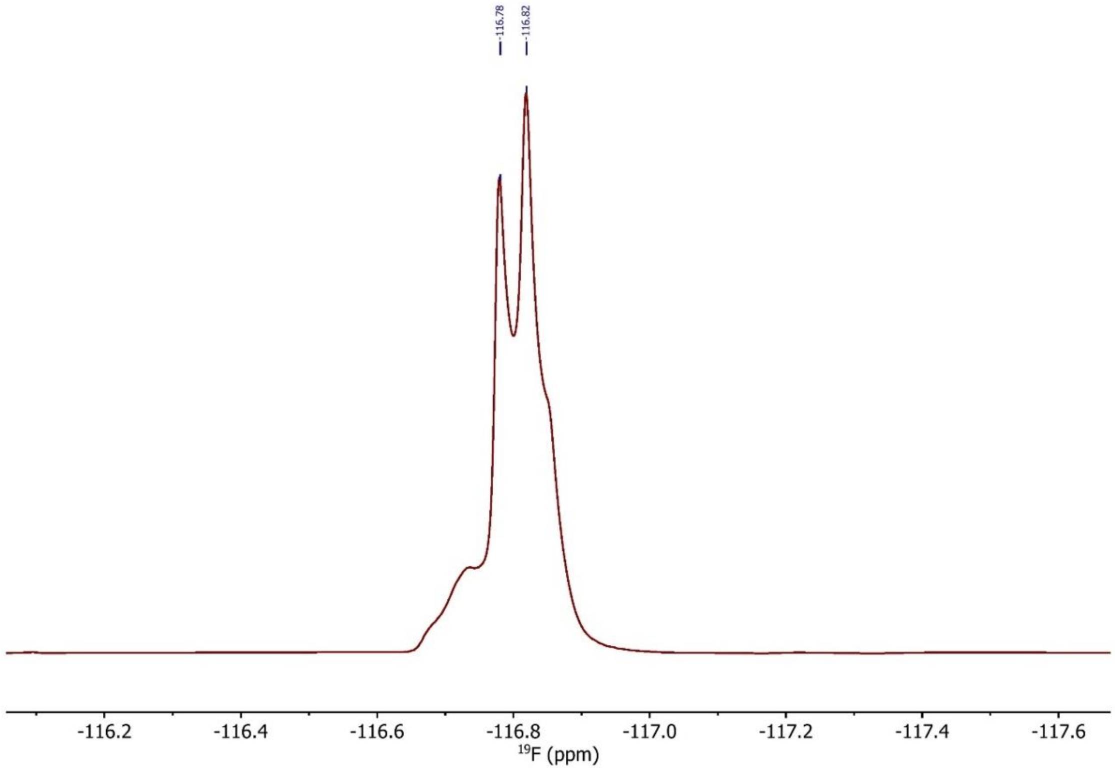

Figure S37. 470.7 MHz $^{19}F\{^{11}B\}$ NMR spectrum of compound **6** in PS/THF-d_8 after seven days of swelling. The splitting corresponds to $^2D_{F\text{-}F}$ = 18.0 Hz. The broad shoulder on the left side of the signal belongs to the ^{10}B isotopologue.

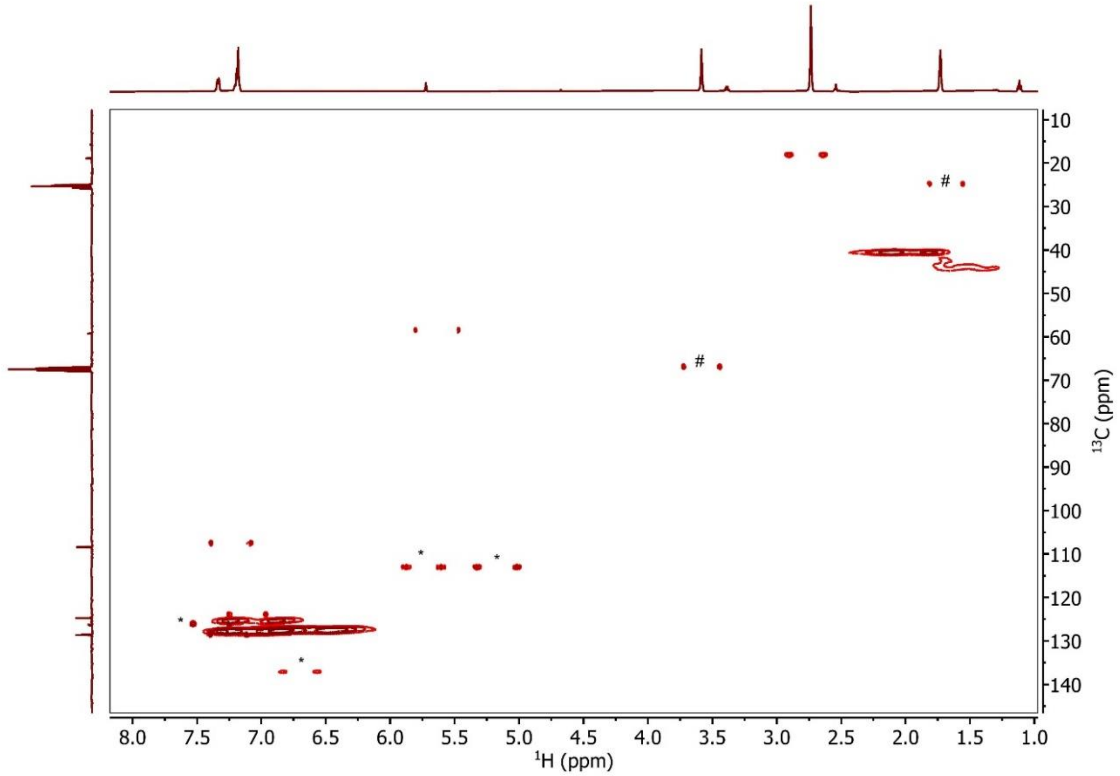

Figure S38. 500.3/125.8 MHz ^{1}H,^{13}C CLIP-HSQC spectrum of compound **6** in PS/THF-d_8 after seven days of swelling. Residual solvent signals are marked with #. Signals marked with * belong to unpolymerized styrene. The broad signals between δ(^{13}C) = 40-50 ppm and 125-130 ppm are from polystyrene. The 1D traces (^{1}H and ^{13}C) were taken from the isotropic spectra.

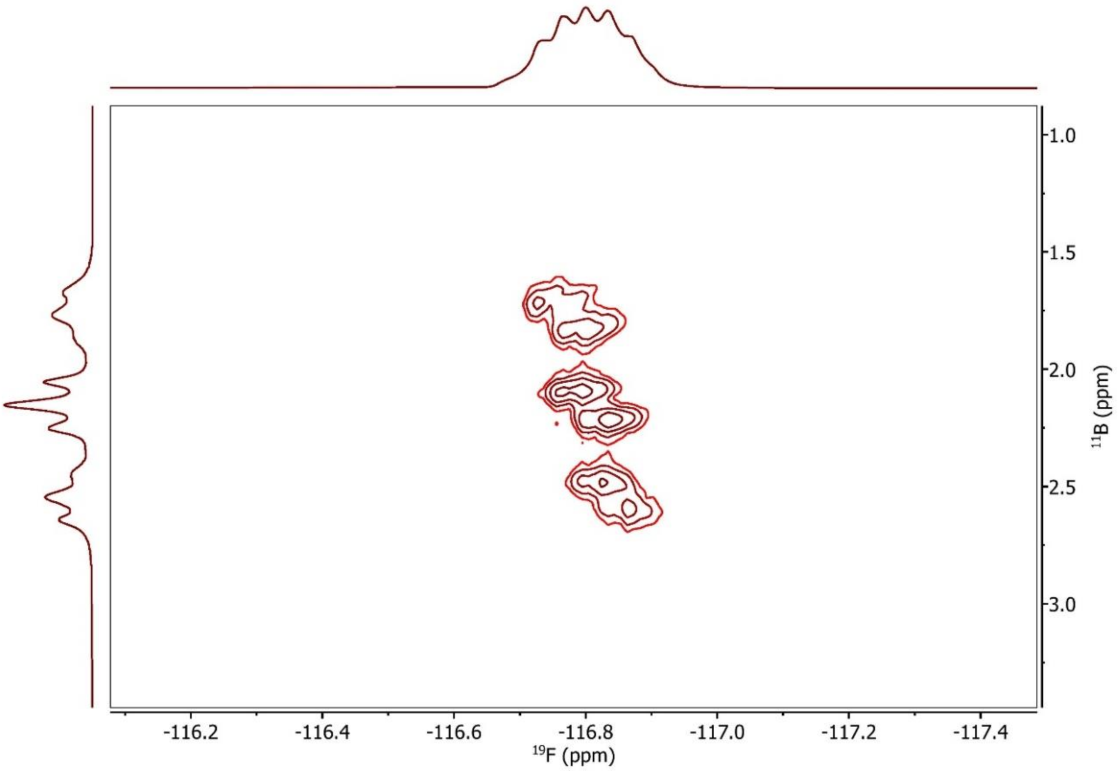

Figure S39. 470.7/160.5 MHz ^{19}F,^{11}B HMQC spectrum of compound **6** in PS/THF-d_8 after seven days of swelling with a ^{11}B flip angle of 30°. From the tilt of the signal, a positive ^{11}B RQC can be deduced.

Alignment tensors

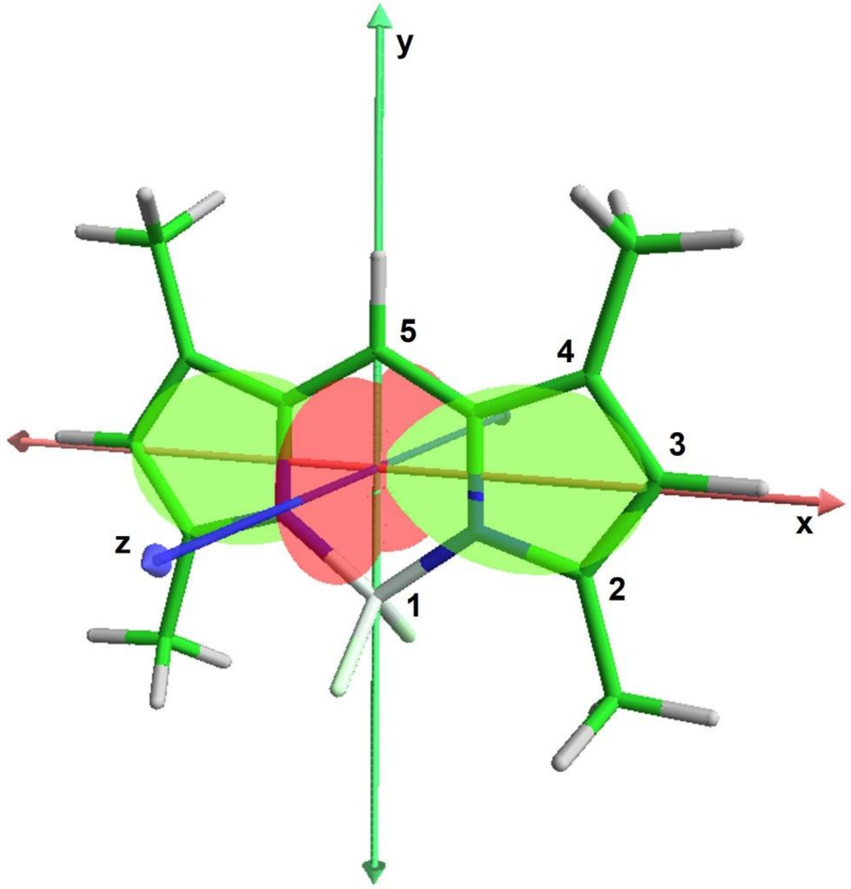

Figure S40. Graphical representation of the alignment tensor (obtained from five one-bond RDCs) for compound **2** in the molecular coordinate system with the positions numbered. Green and red lobes correspond to directions of positive and negative alignment, respectively. Principal values, given in the molecular coordinate system, are A_{xx} = 6.388·10^{-4}, A_{yy} = –1.718·10^{-4} and A_{zz} = –3.669·10^{-4}. The Cornilescu quality factor[11] is 0.021.

Table S4. Experimental chemical shifts (in ppm, determined from the isotropic sample) and one-bond coupling constants (in Hz, where $T = J + D$) obtained from a sample of compound **2** in PS/THF-d$_8$ after seven days of swelling. The experimental ^{11}B RQC is –41.6 Hz, the calculated value is –40.5 Hz. Numbering of the positions is according to **Figure S**40.

Position	δ(^{1}H) (ppm)	δ(^{13}C) (ppm)	$^1J_{C\text{-}H}$ (Hz)	$^1T_{C\text{-}H}$ (Hz)	$^1D_{C\text{-}H,exp}$ (Hz)	$^1D_{C\text{-}H,calc}$ (Hz)
1	-147.2$^{a)}$	0.8$^{b)}$	–32.6$^{c)}$	–19.8$^{c)}$	12.8$^{c)}$	12.2$^{c)}$
2-Me	2.46	14.6	128.7	126.1	–2.6	–3.1
3	6.07	119.4	170.7	132.0	–38.7	–39.9
4-Me	2.25	11.0	127.5	124.9	–2.6	–2.6
5	7.33	121.7	161.2	174.0	12.8	12.3

a) The chemical shift of the fluorine atoms is given here.
b) The chemical shift of the boron atom is given here.
c) The one-bond coupling between fluorine and boron is given here.

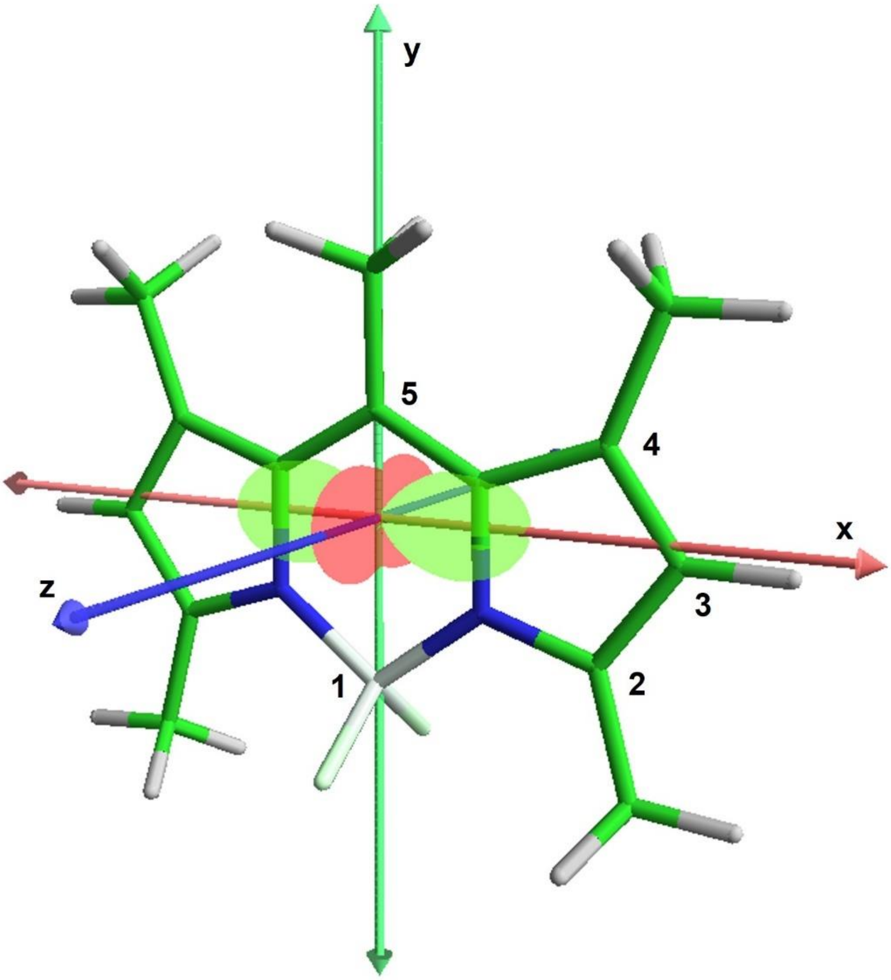

Figure S41. Graphical representation of the alignment tensor (obtained from five one-bond RDCs) for compound **3** in the molecular coordinate system with the positions numbered for discussion. The green and red lobes correspond to directions of positive and negative alignment, respectively. Principal values, given in the molecular coordinate system, are $A_{xx} = 2.898 \cdot 10^{-4}$, $A_{yy} = -9.114 \cdot 10^{-5}$ and $A_{zz} = -1.986 \cdot 10^{-4}$. The Cornilescu quality factor is 0.041.

Table S5. Experimental chemical shifts (in ppm, determined from the isotropic sample) and one-bond coupling constants (in Hz, where *T* = *J* + *D*) obtained from a sample of compound **3** in PS/THF-d_8 after seven days of swelling. The experimental ^{11}B RQC is –37.6 Hz, the calculated value is –37.9 Hz. Numbering of the positions is according to **Figure S**41.

Position	δ(^{1}H) (ppm)	δ(^{13}C) (ppm)	$^1J_{C\text{-}H}$ (Hz)	$^1T_{C\text{-}H}$ (Hz)	$^1D_{C\text{-}H,exp}$ (Hz)	$^1D_{C\text{-}H,calc}$ (Hz)
1	–147.4[a)]	0.5[b)]	–32.5[c)]	–25.5[c)]	7.0[c)]	6.5[c)]
2-Me	2.44	14.2	128.7	127.4	–1.3	–1.7
3	6.07	121.4	170.6	149.9	–20.7	–20.9
4-Me	2.41	17.0	127.6	126.3	–1.3	–0.7
5-Me	2.61	16.4	128.8	126.3	–2.5	–2.1

a) The chemical shift of the fluorine atoms is given here.
b) The chemical shift of the boron atom is given here.
c) The one-bond coupling between fluorine and boron is given here.

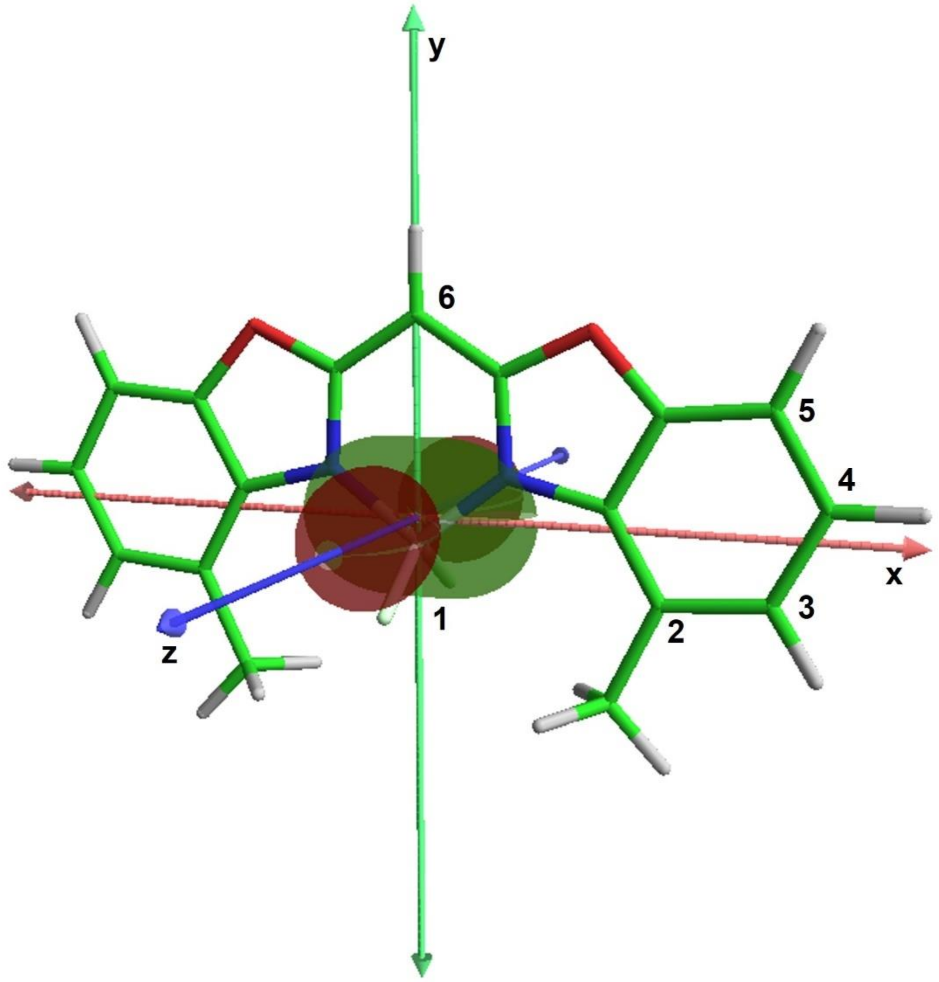

Figure S42. Graphical representation of the alignment tensor (obtained from five one-bond RDCs) for compound **6** in the molecular coordinate system with the positions numbered for discussion. The green and red lobes correspond to directions of positive and negative alignment, respectively. Principal values, given in the molecular coordinate system, are $A_{xx} = 2.760 \cdot 10^{-4}$, $A_{yy} = 1.793 \cdot 10^{-4}$ and $A_{zz} = -4.553 \cdot 10^{-4}$. The Cornilescu quality factor is 0.048.

Table S6. Experimental chemical shifts (in ppm, determined from the isotropic sample) and one-bond coupling constants (in Hz, where $T = J + D$) obtained from a sample of compound **6** in PS/THF-d$_8$ after seven days of swelling. The experimental ^{11}B RQC is 63.0 Hz, the calculated value is 50.3 Hz. Numbering of the positions is according to **Figure S**42.

Position	$\delta(^1H)$ (ppm)	$\delta(^{13}C)$ (ppm)	$^1J_{C\text{-}H}$ (Hz)	$^1T_{C\text{-}H}$ (Hz)	$^1D_{C\text{-}H,exp}$ (Hz)	$^1D_{C\text{-}H,calc}$ (Hz)
1	–117.1$^{a)}$	2.1$^{b)}$	–27.1$^{c)}$	–15.8$^{c)}$	11.3$^{c)}$	10.3$^{c)}$
2-Me	2.72	18.9	128.3	132.7	4.4	3.8
3	7.17	128.6	159.4	$^{d)}$	$^{d)}$	-
4	7.18	124.7	163.9	144.4	–19.5	–19.8
5	7.33	108.3	168.7	155.0	–13.7	–14.3
6	5.71	59.2	179.9	166.5	–13.4	–13.1

a) The chemical shift of the fluorine atoms is given here.
b) The chemical shift of the boron atom is given here.
c) The one-bond coupling between fluorine and boron is given here.
d) No value could be obtained for this position due to signal overlap with the polystyrene.

Interference of quadrupolar and dipolar frequency shifts in **6**

For the m_B = (–1/2→ –3/2) (transitions A-C) we obtain from the calculated EFG:

$$\boldsymbol{\Delta\nu_Q} = \frac{9.673\text{MHz}}{2\text{ a.u.}}\begin{pmatrix} V_{xx} & 0 & 0 \\ 0 & V_{yy} & 0 \\ 0 & 0 & V_{zz} \end{pmatrix} = \begin{pmatrix} 59.1 & 0 & 0 \\ 0 & 12.3 & 0 \\ 0 & 0 & -71.4 \end{pmatrix}\text{kHz}$$

With (r = 1.389 Å, θ = 55.65 °) we obtain for transitions A-C (m_F = ++, +-, --, respectively)

$$\boldsymbol{\Delta\nu_D}(A) = \frac{27.06\text{ kKz}}{2}\begin{pmatrix} -1 & 0 & 0 \\ 0 & -0.045 & 0 \\ 0 & 0 & 1.045 \end{pmatrix} = \begin{pmatrix} -13.5 & 0 & 0 \\ 0 & -0.6 & 0 \\ 0 & 0 & 14.1 \end{pmatrix}\text{kHz}$$

$$\boldsymbol{\Delta\nu_D}(B) = \frac{27.06\text{ kKz}}{2}\begin{pmatrix} 0 & 0 & 0 \\ 0 & 0 & 1.398 \\ 0 & 1.398 & 0 \end{pmatrix} = \begin{pmatrix} 0 & 0 & 0 \\ 0 & 0 & 18.9 \\ 0 & 18.9 & 0 \end{pmatrix}\text{kHz}$$

$$\boldsymbol{\Delta\nu_D}(C) = \frac{27.06\text{ kKz}}{2}\begin{pmatrix} 1 & 0 & 0 \\ 0 & 0.045 & 0 \\ 0 & 0 & -1.045 \end{pmatrix} = \begin{pmatrix} 13.5 & 0 & 0 \\ 0 & 0.6 & 0 \\ 0 & 0 & -14.1 \end{pmatrix}\text{kHz}$$

Hence, the sum of $\boldsymbol{\Delta\nu_Q}$ and $\boldsymbol{\Delta\nu_D}$ for transitions A-C is given by:

$$\boldsymbol{\Delta\nu_Q} + \boldsymbol{\Delta\nu_D}(A) = \begin{pmatrix} 45.6 & 0 & 0 \\ 0 & 11.7 & 0 \\ 0 & 0 & -57.3 \end{pmatrix}\text{kHz}$$

$$\boldsymbol{\Delta\nu_Q} + \boldsymbol{\Delta\nu_D}(B) = \begin{pmatrix} 59.1 & 0 & 0 \\ 0 & 12.3 & 18.9 \\ 0 & 18.9 & -71.4 \end{pmatrix}\text{kHz}$$

$$\boldsymbol{\Delta\nu_Q} + \boldsymbol{\Delta\nu_D}(C) = \begin{pmatrix} 72.6 & 0 & 0 \\ 0 & 12.9 & 0 \\ 0 & 0 & -85.5 \end{pmatrix}\text{kHz}$$

The magnitudes are roughly in a ratio 4 : 5 : 6.

With a ~20% larger EFG (from the experimental ^{11}B RQC) the magnitudes would be in a ratio 5 : 6 : 7.

Relaxation times

Table S7. T_1 relaxation times for ^{11}B (128 MHz) and ^{19}F (376 MHz) of **2**, **3** and **6** in PS/THF-d_8.

	**2**	**3**	**6**
T_1 (^{11}B) (ms)	410 ± 10	190 ± 10	100 ± 10
T_1 (^{19}F) (ms)	680 ± 10	710 ± 10	440 ± 10

Further structures

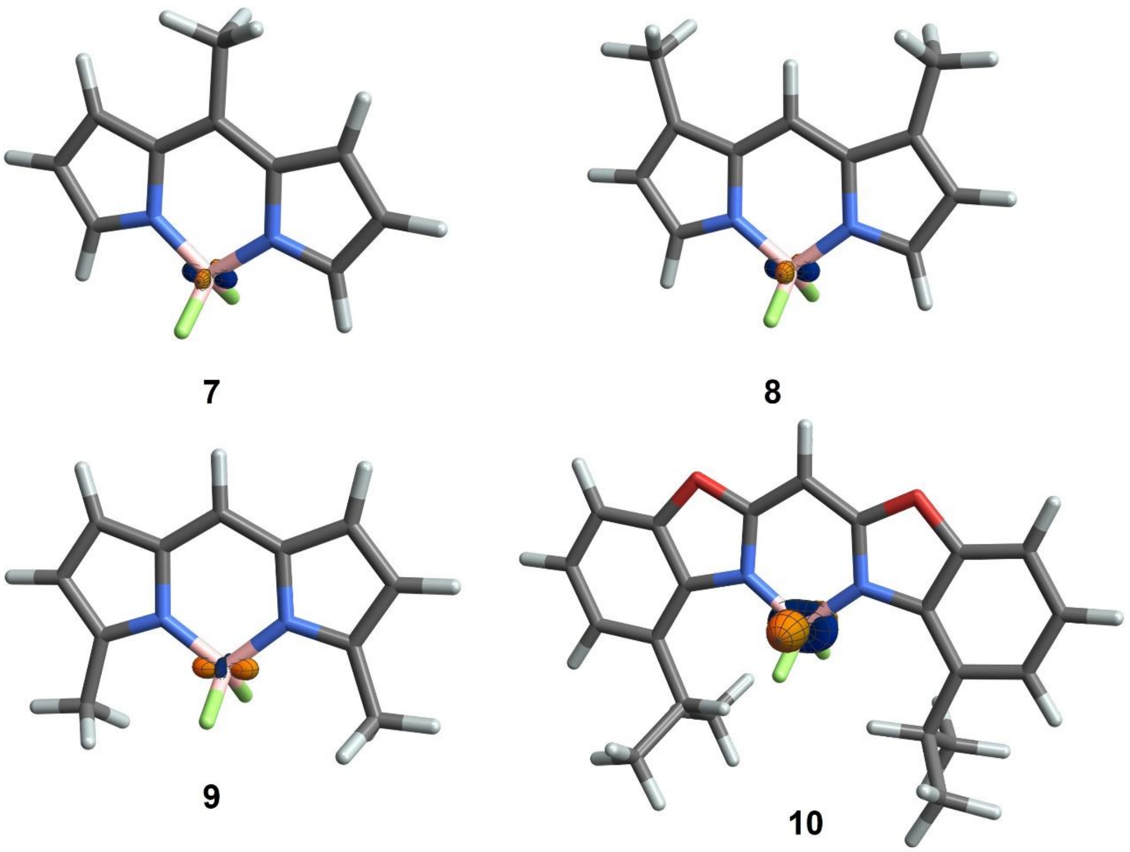

Figure S43. Geometry-optimized structures **7**-**10**. Boron EFG tensors are shown with the same scaling factor as in Figure 1.

Table S8. Selected bond lengths (BN, BF) and angles (NBN, FBF) in geometry-optimized structures **7**-**10**.

	**7**	**8**	**9**	**10**
BN (Å)	1.562	1.566	1.563	1.565
BF (Å)	1.386	1.387	1.393	1.389/1.392 [a]
NBN (°)	105.4	105.7	106.7	106.8
FBF (°)	111.0	110.9	110.4	111.4

[a] due to butterfly folding, the two fluorine atoms become inequivalent

Table S9. EFG components in geometry-optimized structures **7**-**10**.

	**7**	**8**	**9**	**10**
V_{xx} (10^{-3} a.u.) [a]	5.8	6.0	–6.8	7.7
V_{yy} (10^{-3} a.u.) [a]	–0.6	0.5	2.2	5.2 [b]
V_{zz} (10^{-3} a.u.) [a]	–5.2	–6.4	4.6	–13.0 [b]

[a] 1 a.u. = 9.717×10^{-21} Vm^{-2}.

[b] due to butterfly folding, the off-diagonal element $V_{yz} = 1.9 \times 10^{-3}$ a.u. becomes nonzero.

References

[1] X. Wang, F. Rüttger, J. Kretsch, A. Kreyenschmidt, R. Herbst-Irmer, D. Stalke, *Dalton Trans.* **2024**, *53*, 8264.

[2] A. Enthart, J. C. Freudenberger, J. Furrer, H. Kessler, B. Luy, *J. Magn. Reson.* **2008**, *192*, 314.

[3] A.-C. Pöppler, S. Frischkorn, D. Stalke, M. John, *ChemPhysChem* **2013**, *14*, 3103.

[4] F. Rüttger, D. Stalke, M. John, *Chem. Commun.* **2023**, *59*, 14657.

[5] A. Navarro-Vázquez, *Magn. Reson. Chem.* **2012**, S73.

[6] M. J. Frisch, G. W. Trucks, H. B. Schlegel, G. E. Scuseria, M. A. Robb, J. R. Cheeseman, G. Scalmani, V. Barone, G. A. Petersson, H. Nakatsuji, X. Li, M. Caricato, A. V. Marenich, J. Bloino, B. G. Janesko, R. Gomperts, B. Mennucci, H. P. Hratchian, J. V. Ortiz, A. F. Izmaylov, J. L. Sonnenberg, D. Williams-Young, F. Ding, F. Lipparini, F. Egidi, J. Goings, B. Peng, A. Petrone, T. Henderson, D. Ranasinghe, V. G. Zakrzewski, J. Gao, N. Rega, G. Zheng, W. Liang, M. Hada, M. Ehara, K. Toyota, R. Fukuda, J. Hasegawa, M. Ishida, T. Nakajima, Y. Honda, O. Kitao, H. Nakai, T. Vreven, K. Throssell, J. A. Montgomery, Jr., J. E. Peralta, F. Ogliaro, M. J. Bearpark, J. J. Heyd, E. N. Brothers, K. N. Kudin, V. N. Staroverov, T. A. Keith, R. Kobayashi, J. Normand, K. Raghavachari, A. P. Rendell, J. C. Burant, S. S. Iyengar, J. Tomasi, M. Cossi, J. M. Millam, M. Klene, C. Adamo, R. Cammi, J. W. Ochterski, R. L. Martin, K. Morokuma, O. Farkas, J. B. Foresman, and D. J. Fox, *Gaussian16*, Gaussian, Inc., Wallingford CT, **2016**.

[7] a) F. Weigend, R. Ahlrichs, *Phys. Chem. Chem. Phys.* **2005**, *7*, 3297; b) F. Weigend, *Phys. Chem. Chem. Phys.* **2006**, *8*, 1057.

[8] a) S. Grimme, *J. Comput. Chem.* **2006**, *27*, 1787; b) S. Grimme, J. Antony, S. Ehrlich, H. Krieg, *J. Chem. Phys.* **2010**, *132*, 154104; c) S. Grimme, S. Ehrlich, L. Goerigk, *J. Comput. Chem.* **2011**, *32*, 1456.

[9] M. D. Hanwell, D. E. Curtis, D. C. Lonie, T. Vandermeersch, E. Zurek and G. R. Hutchison, *J. Cheminf.* **2012**, *4*, 17.

[10] a) E. Zurek, C. J. Pickard, J. Autschbach, *J. Phys. Chem. C* **2008**, *112*, 11744; b) J. Autschbach, S. Zheng, R. W. Schurko, *Concepts Magn. Reson. Part A* **2010**, *36A*, 84.

[11] G. Cornilescu, J. L. Marquardt, M. Ottiger, A. Bax, *J. Am. Chem. Soc.* **1998**, *120*, 6836.
